# Highly Effective Adsorption Process of Ni(II) Ions with the Use of Sewage Sludge Fly Ash Generated by Circulating Fluidized Bed Combustion (CFBC) Technology

**DOI:** 10.3390/ma14113106

**Published:** 2021-06-05

**Authors:** Tomasz Kalak, Kinga Marciszewicz, Joanna Piepiórka-Stepuk

**Affiliations:** 1 Department of Industrial Products and Packaging Quality, Institute of Quality Science, Poznań University of Economics and Business, Niepodległości 10, 61-875 Poznań, Poland; kmarciszewicz@gmail.com; 2 Department of Mechanical Engineering, Division of Food Industry Processes and Facilities, Koszalin University of Technology, Racławicka 15-17, 75-620 Koszalin, Poland; joanna.piepiorka@tu.koszalin.pl

**Keywords:** water quality, waste management, removal efficiency, sewage sludge fly ash, Ni(II) ions, kinetics

## Abstract

Recently, more and more attention has been paid to the removal of nickel ions due to their negative effects on the environment and human health. In this research, fly ash obtained as a result of incineration of municipal sewage sludge with the use of circulating fluidized bed combustion (CFBC) technology was used to analyze the possibility of removing Ni(II) ions in adsorption processes. The properties of the material were determined using analytical methods, such as SEM-EDS, XRD, BET, BJH, thermogravimetry, zeta potential, SEM, and FT-IR. Several factors were analyzed, such as adsorbent dose, initial pH, initial concentration, and contact time. As a result of the conducted research, the maximum sorption efficiency was obtained at the level of 99.9%. The kinetics analysis and isotherms showed that the pseudo-second order equation model and the Freundlich isotherm model best suited this process. In conclusion, sewage sludge fly ash may be a suitable material for the effective removal of nickel from wastewater and the improvement of water quality. This research is in line with current trends in the concepts of circular economy and sustainable development.

## 1. Introduction

The last decades of human activity have generated many environmental problems, such as water pollution (rivers, lakes, etc.), water waste, pollution of seas and oceans with industrial and consumer waste, and pollution with heavy metals and other toxic substances. The frequency of these phenomena is increasing, and despite taking many corrective and preventive measures, it still seems that the processes of environmental pollution are not stopping. It is necessary to take steps globally on many levels, from legal regulations to the appropriate, responsible attitude of every human being. Heavy metals are particularly dangerous to the environment and humans. They are not biodegradable, and they are able to accumulate in the ecosystem through the food chain and accumulate in living organisms. Even in low concentrations, they are carcinogenic, cause diseases of various systems and organs, and can also cause loss of life [1].

Nickel is one of toxic heavy metals that naturally occur in the environment and wastewaters. Nickel compounds are used in various industries (e.g., metal electroplating, silver refineries, storage battery industries, zinc base casting, etc.) that discharge their significant amounts into the environment in various forms, and their concentrations in industrial wastewater range from 3.40 to 900 mg/L [2]. The maximum nickel concentration limit in bottled water was set at 50 mg/L by the European Economic Community [3]. According to the United States Environmental Protection Agency (USEPA), Indian Standards Institution (ISI), and World Health Organization (WHO), the permissible limits of nickel in drinking water are 0.1, 0.05, and 0.02 mg/L, respectively [4,5]. Nickel is one of the ubiquitous elements found in soil, water, atmospheric air, and the biosphere. Nickel enters into the soil via wind and during rainfall. Another source is the combustion of solid fuel (coal, oil) and waste, as well as industrial processes. An excessive amount of metal in plants may result in the appearance of phytotoxic symptoms, such as reduced photosynthesis, growth inhibition, and changes in enzyme activity. In addition, it can cause biochemical and physiological stress in plants, accompanied by the formation of hydrogen peroxide. This compound prevents the cells from working properly. Plant stress is a reaction to unfavorable environmental conditions. It is possible to develop a disease called chlorosis, during which the process of producing chlorophyll is disturbed, and diseased plants turn yellow. The growth of roots is inhibited, and they turn brown and thicken. In addition, nickel in green parts of plants disturbs the cation–anionic balance, which is responsible for good plant growth and development. In extreme conditions, it can cause plants to die [6].

Nickel exposure occurs mainly through the inhalation of dust and vapor particles. An excess of an element in the human body can accumulate in the lymph nodes, causing changes in the bone marrow and chromosomes. The high concentration of nickel sulphide they contain can cause serious damage to lungs and kidneys. On the other hand, prolonged exposure to dust in the worst cases leads to asthma, bronchitis, and pneumoconiosis. The most toxic nickel-containing compound is nickel carbonyl. Its excess in the human body can lead to pain, dizziness, insomnia, or vomiting. The respiratory system and most organs such as kidneys, liver, spleen, and brain are damaged. High levels of nickel in the human body can cause lung and bone cancer [7].

In everyday life, the widespread use of items containing nickel poses a great risk of contact allergy. This highly sensitizing metal can lead to irritation or contact eczema accompanied by burning and itchy skin. The metal is the most common allergen. Hypersensitivity can appear at any age. Everyday items are not the only source of allergies. Nickel found in food, tap water, and groundwater causes contact eczema and other chronic skin diseases (e.g., dermatopathy). The symptoms are very similar to those caused by food allergies [7].

Many advanced technologies are used to treat wastewater from heavy metals, such as ion exchange, ultrafiltration, reverse osmosis, chemical precipitation, electrolysis, and more [8,9]. Due to high material and operating costs, other alternative technologies are sought, as well as cheaper solutions and materials. These include adsorption processes, which have become more and more popular in recent years. They are characterized by low operating and equipment costs, ease of use, and the possibility of application to various types of contamination. The advantages include the possibility of using cheap industrial wastes as adsorbents, including fly ash obtained from the incineration of various wastes and materials, including municipal sewage sludge [10,11,12,13,14].

One of the most effective techniques for neutralizing municipal sewage sludge is thermal treatment, which brings many economic, energy, and environmental benefits and is recommended by European Union legislation. The quantity and quality of fly ash (FA) is dependent on the chemical composition of sludge, incineration, and exhaust gas treatment technologies [15]. The calorific value of average sewage sludge with 80% hydration is about 0.5 MJ/kg. The calorific value of dust with a moisture content below 20% is between 12 and 18 MJ/kg, and the bulk density is approx. 700 kg/m^3^ [16]. In addition, this material is odorless, stable, safe in terms of sanitation, free from pathogenic organisms, non-biodegradable, and not harmful to human health and the environment. Fly ash resulting from fluidization combustion usually contains elements such as Ca, Cu, Mg, Mn, K, P, Zn, B, Mo, and Fe, which are useful, for example, for plant growth. Fly ash can be a potential raw material for many industrial applications where there is a need for its binding properties and favorable chemical and physical properties. Important characteristics such as particle size, porosity, bulk density, water holding capacity, and surface area make FA suitable for the use as an adsorbent for many metal ions and organic and inorganic compounds. Some of the physicochemical properties of FA are changed as a result of frequent changes in the composition of municipal wastewater subjected to combustion processes. As a result, its industrial application may also change [17,18].

Most modern technologies of thermal treatment of municipal sewage sludge include circulating fluidized bed combustion (CFBC) technology. The main advantages of the technology are as follows: compatibility with many types of fuels, low emission of pollutants, high process efficiency, ease of maintenance, and the possibility of simultaneous combustion of dehydrated, dried, and fermented sludge. Many processes are involved in sewage sludge combustion, such as sewage sludge drying, combustion in the circulating fluidized bed furnace at a high temperature of 650 °C that produces waste gases and slag, cooling of gases, purification of gases in semi-dry and dry reactors using lime milk, active carbon, hydrated lime and ammonia, final purification of gases using bag filters, and the obtaining of fly ash.

About 20–25% of municipal solid waste produced in the European Union (EU) is treated by incineration. In 2020, the total amount of sewage sludge production was estimated at about 13 million tons, and there were 3664 large combustion plants in the EU (EU-28, including Great Britain before leaving the EU). Germany (1794.443 thousand tonnes in 2016), Spain (1174.4 thousand tonnes in 2016), France (1174 thousand tonnes in 2017), Poland (583.07 thousand tonnes in 2018), Turkey (318.503 thousand tonnes in 2018), and Romania (247.76 thousand tonnes in 2018) are the greatest producers of sewage sludge in the EU. The average mass of municipal solid waste incinerated is just under 200,000 tonnes per year. In Poland, there are several mono-incineration plants located in such cities as Warsaw, Kraków, Gdańsk, Gdynia, Bydgoszcz, Łódź, Olsztyn, Szczecin, Kielce, Łomża, and Zielona Góra [19,20,21,22].

This huge amount of produced sewage sludge should be disposed of, and an alternative may be incineration to reduce weight of waste and generate FA. One of the potential applications of FA from sewage sludge incineration may be in adsorption processes to remove metal ions from wastewater and to improve the purity of aquatic environment and water quality.

The goal of this investigation was to examine the adsorption process of Ni(II) ions in aqueous solutions using fly ash generated from the combustion of municipal sewage sludge in the circulating fluidized bed combustion (CFBC) technology under different conditions of initial and equilibrium pH, adsorbent dosage, initial concentration, and contact time. Furthermore, the aim was to determine properties of the adsorbent material and to estimate the process kinetics, equilibrium, and isotherms.

## 2. Experimental Procedure

### 2.1. Materials and Methods

#### 2.1.1. Sewage Sludge Fly Ash Preparation

The sewage sludge fly ash (SS-FA) used in these studies was generated in one of the plants located in Poland (Greater Poland voivodeship) in the process of burning municipal sewage sludge in the Sludge Thermal Transformation Installation using CFBC technology (Bydgoszcz, Poland). Four samples of fly ash were collected from different places in the ash container (silo) after all stages of purification processes in the CFBC installation, and next combined into one sample and mixed. Then fly ash was sieved to separate different grain fractions, and particles of less than 0.212 mm were used in the experiments. Samples were dried at 105 °C to constant weight, and finally the moisture content was less than 0.2%. In these studies, all chemicals were analytically pure, and distilled water was used.

#### 2.1.2. Sewage Sludge Fly Ash Characterization

In the first stage, the sewage sludge fly ash (SS-FA) was analyzed for its physicochemical properties with the use of various techniques: bulk density, granulation analysis, SEM-EDS analysis, XRD, pore volume and specific surface area (BET, BJH), zeta potential, TGA analysis, SEM morphology, and FT-IR. The research methods and apparatus used were described in detail and included as Supplementary Material (Methods).

#### 2.1.3. Nickel(II) Adsorption Process

The adsorption experiments of Ni(II) ions in aqueous solutions were carried out at room temperature (23 ± 1 °C) and under normal pressure conditions. The solutions of Ni(II) (nickel standard for AAS in nitric acid (HNO_3_), pure for analysis) were used. The SS-FA samples (weight from 2.5 to 500 mg/L) and Ni(NO_3_)_2_ solution (20 mL, initial concentration 20–100 mg/L) were mechanically shaken in conical flasks at 200 rpm (1 h). The pH of Ni(II) initial solutions was set with 0.1 M NaOH and HNO_3_. Subsequently, the SS-FA and solutions after adsorption processes were centrifuged at 4000 rpm for phase separation. Next, the concentration of Ni(II) ions (mg/L) was determined with the use of SpectrAA 800 spectrophotometer (F-AAS, wavelength λ = 232 nm for nickel Ni, Varian, Palo Alto, USA). The measurements were repeated three times, and calculated arithmetic mean values were finally shown in the results.

The adsorption efficiency *A* (%) and adsorption capacity *q_e_* (mg/g) were estimated based on Formulas (1) and (2), respectively:

(1) $$A=\left[\frac{C_{0}-C_{e}}{C_{0}}\right]\times 100\%$$

(2) $$q_{e}=\frac{\left(C_{0}-C_{e}\right)\times V}{m}$$

where *C*_0_ and *C_e_* (mg/L) are initial and equilibrium Ni(II) ion concentrations, respectively; *V* (L)—volume of solution, m (g)—mass of SS-FA.

Pseudo-first-order, pseudo-second-order kinetic models, and Langmuir and Freundlich adsorption models were estimated based on Formulas (3)–(6), respectively:

(3) $$q_{t}=q_{e}\left(1-e^{k_{1}t}\right)$$

(4) $$q_{t}=\frac{q_{e}^{2}k_{2}t}{1+q_{e}k_{2}t}$$

(5) $$q_{e}=\frac{q_{max}K_{L}C_{e}}{1+K_{L}C_{e}}$$

(6) $$q_{e}=K_{F}C_{e}^{\frac{1}{n}}$$

where *q_e_* (mg/g)—the maximum amount of Ni(II) ions adsorbed per mass of SS-FA at equilibrium; *k*_1_ (1/min)—the rate constant of pseudo-first-order adsorption; *k_2_* (g/(mg·min.))—the rate constant of pseudo-second-order adsorption, *K_L_*—the Langmuir constant; *q_max_* (mg/g)—the maximum adsorption capacity; *C_e_* (mg/L)—the equilibrium concentration after the adsorption process; *1/n*—the intensity of adsorption; *K_F_*—the Freundlich constant, *q_t_* (mg/g)—the amount of Ni(II) ions adsorbed at any time *t* (min).

## 3. Results and Discussion

### 3.1. Characterization of the SS-FA Adsorptive Material

Fly ash resulting from the combustion of municipal sewage sludge was characterized by many methods. Firstly, the granulation analysis showed the following grain fractions: < 0.212 mm (90.75 ± 1.3%), 0.212–0.5 mm (8.45 ± 1.2%), 0.5–0.71 mm (0.49 ± 0.07%), 0.71–1.0 mm (0.31 ± 0.06%), 1.0–1.7 mm (0%), >1.7 mm (0%). Many researchers indicated in the literature that smaller FA particle sizes resulted in higher metal ion removal efficiency. This is due to the presence of larger specific surface area and the number of active centers capable of adsorbing metal ions [23]. Hence, following the literature analyses and research results, it can be concluded that it is appropriate to use SS-FA particles with the smallest particle size range to carry out Ni(II) adsorption experiments [24,25,26,27].

The SS-FA particles are irregular in shape and size, and often form various agglomerates, which affects the density of the bulk material. Therefore, bulk density of the SS-FA loose grains was first determined and was equal to 0.83 ± 0.03 g/cm^3^. Then, the material sample was compacted on a vibrating table, and the following density was obtained: 1.56 ± 0.04 g/cm^3^. Similar results were reported in the literature [28,29]. These analysis results may be relevant for the possible use of SS-FA as an additive to ceramic, construction, or building products.

**Figure 1:**
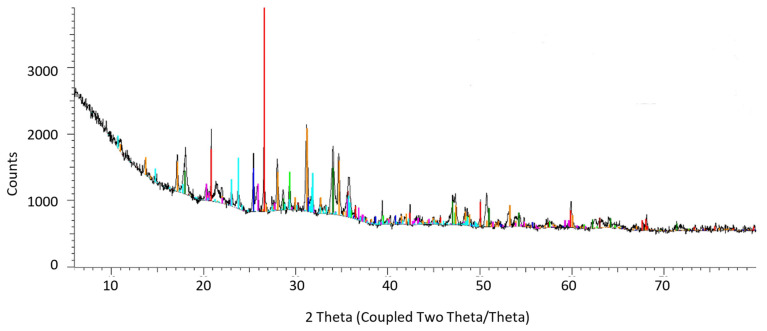
X-ray diffraction spectrum of SS-FA.

X-ray diffraction analysis of SS-FA was carried out, and the spectrum is presented in Figure 1. The following chemical compounds have been shown to be the main crystalline phases of SS-FA: calcium sulfate (CaSO_4_, 6%), maghemite (synFe_2_O_3_, 6.38%), magnesium phosphate (Zn_2_Mg(PO_4_)*_2_*, 6.75%), calcite (CaCO_3_, 9.25%), portlandite (Ca(OH)_2_, 11.4%), quartz (synSiO_2_,13.16%), whitlockite (Ca2.59 MgO 41 (PO_4_)_2_, 16.83%), and stanfieldite (synMg_3_Ca_3_(PO_4_)_4_, 30.23%). It should be noted here that SS-FA is a heterogeneous multi-component product of the combustion process. Therefore, depending on the place of sampling in the silo tank, the quantitative compositions may slightly differ. However, X-ray diffraction analysis should reveal the same crystalline phases of the substances. Similar results of XRD analysis have been found in the literature [30,31,32].

Measurements of particle size distribution were carried out, and the results are attached as Supplementary Material (Figure S1). It should be mentioned that during the analysis the larger and heavier SS-FA particles fell to the bottom of the suspension, and the apparatus measured the smaller and lighter particles that remained in suspension. Therefore, as a result of the measurement, one peak was observed with a particle size of 1205 nm. Other authors obtained similar results on different fly ash samples [33,34]. According to the literature, smaller particles have the ability to dissolve or suspend in solutions more quickly and form a more stable suspension compared to larger ones. Additionally, smaller particles obtain higher adsorption in metal ion binding processes [24,35].

**Figure 2:**
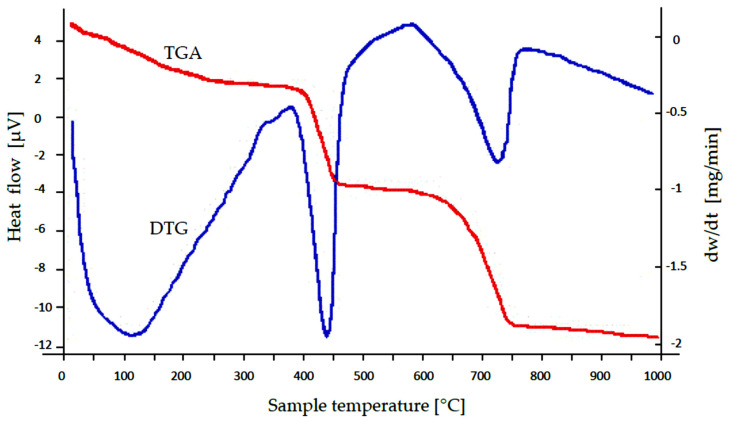
Thermogravimetric curves of SS-FA.

Thermogravimetric analysis was performed, and the results are presented in Figure 2. Generally speaking, a weight loss of the sample was observed with increasing temperature up to 1000 °C. The TGA curve represents the change in weight loss, and the DTG curve represents a derivative weight loss *dw*/*dt* (mg/min) as a function of temperature. Initial weight loss up to 400 °C may be due to evaporation of moisture, volatile organic compounds, and removal of carbon monoxide. Significant weight loss was observed at 400–450 °C (an endothermic peak), which may result from the dehydroxylation reaction of calcium hydroxide [36,37]. A further increase in temperature causes another loss of SS-FA mass at 750 °C. This phenomenon may be a consequence of the degradation of the grain wall surfaces caused by the release of gaseous products (CO_2_) from their interior according to reaction Equation (7) [35,38,39]. Based on the literature, the presence of such CaCO_3_ polymorphs as aragonite, vaterite, and calcite can be assumed [40]. In these studies, the XRD analysis confirmed the presence of calcite (9.25%). Other possible gases may be CO, H_2_, and CH_4_ as a result of decomposition of fly ash particles [41].

CaCO_3_ → CaO + CO_2_ (7)

**Figure 3:**
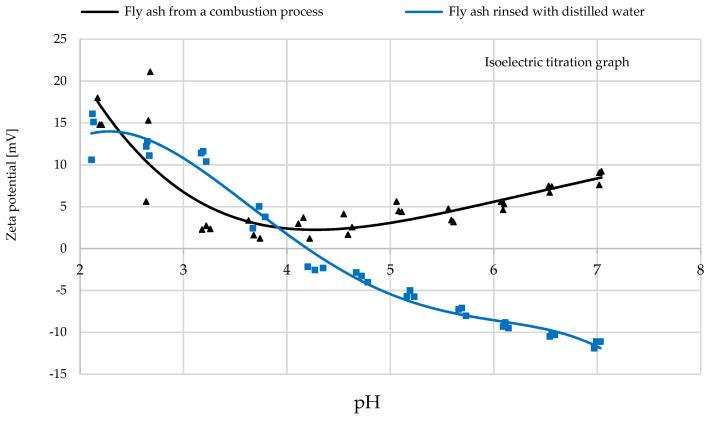
Plots of zeta potential vs. equilibrium pH.

The analysis of the electrokinetic zeta potential was carried out on the SS-FA before and after rinsing with distilled water (Figure 3). The zeta potential is an important parameter in determining the stability of a solution and indicates the variability of surface potential for a specific material. It was found out that the surface charge of SS-FA varies with the pH of the solution and decreases from 21.2 mV (pH 2.6) to 1.3 mV (pH 4.3) and then increases to 9.3 (pH 7). In the range of pH 2.0–2.7, low stability is a feature of this system. The charge does not reach the isoelectric point (IEP), which means that the surface charge was positive. This non-monotonic variation in fly ash as a function of pH is reported due to the possible dipolar interaction. An increase in pH can reduce the mobility of bridging protons, thus increasing the number of acid sites. This may be associated with the formation of Brønsted acid sites in aluminosilicates. Acid sites are formed with hydroxyl groups located between aluminum and silicon occupied by oxygen tetrahedra. The Si/Al ratio influences the acidity of aluminosilicates in such a way that the total number of acid sites increases as the Si/Al ratio decreases. Replacing Si atoms with Al atoms in the material structure causes a loss of electric charge, which can be neutralized by the presence of an additional cation in the pore structure. Another explanation of non-monotonic behavior may also be due to morphological parameters such as size and shape of aggregates as well as chains formed in SS-FA particles. Additionally, the particle and pore size as well as *S_BET_* value affect the amount of bound water with less activity acting as a solvent. In turn, water can affect the mobility of protons and the structure of the electrical double layer (EDL). The EDL should be different with the same surface structure in pores of the adsorbent and on its surface of micro- and nanoparticles. Parameters such as pH, temperature, and solution composition can have impacts on the structure of the EDL on the particle surface and in pores of different sizes [42,43,44,45,46,47]. In the next step, the SS-FA was rinsed with distilled water in order to obtain a pH of 7 of the dispersion system. This procedure changed the value of the surface charge from positive (16.0 mV, pH 2.2) to negative (–11.2 mV, pH 7). The isoelectric point (IEP) was reached at pH 4.07, which represents the achieved unstable suspension and equilibrium between negative and positive charges. At the IEP point, the SS-FA particles may have the lowest osmotic pressure, viscosity, and solubility.

**Figure 4:**
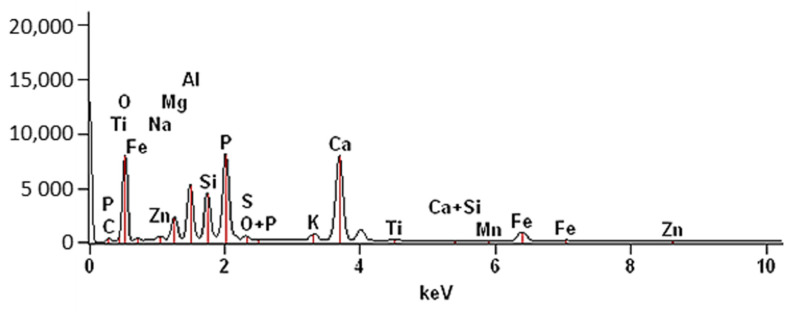
EDS spectrum of SS-FA (magn. x200).

The SEM-EDS analysis was performed, and the results are presented in Figure 4 and Table 1. The plot shows the peaks corresponding to individual elements and oxides, the dominant of which were as follows: O, Ca, P, Al, Si, Fe, Mg and P_2_O_5_, CaO, Al_2_O_3_, SiO_2_, Fe_2_O_3_, and CO_2_, respectively. Other elements and oxides were present in smaller amounts. Table 1 compares the elemental composition with other research results published in the literature. Similar content was observed for Fe_2_O_3_, MnO, Na_2_O, K_2_O, and TiO_2_. The concentrations of other oxides (CaO, SiO_2_, P_2_O_5_, MgO, Al_2_O_3_, SO_3_) were slightly different. Differences may be due to differences in chemical composition, different combustion parameters of the samples, or different analytical techniques.

As a result of BET analysis, adsorption and desorption surface parameters, including specific surface area (*S_BET_*), pore volume of 0.114 cm^3^/g, and average pore diameter (*Apd*), were determined and are presented in Table 2 and Supplementary Material (Figures S2–S4). The shapes of the adsorption isotherms are characteristic of type III isotherms, which are convex towards the pressure axis. The type III isotherm refers to a situation where the adsorbed molecules occur at the most advantageous locations on the surface of a non-porous or macroporous adsorbent. This shape speaks to the so-called cooperative adsorption, which informs that previously adsorbed molecules increase the adsorption of other particles. In the case of low relative pressure, weak interactions between the adsorbate and the adsorbent cause adsorption with low efficiency. When the molecule is adsorbed once, the adsorbate–adsorbate interaction promotes the sorption of more molecules in the adsorption system, as a result of which the isotherm becomes convex towards the pressure axis [54].

**Figure 5:**
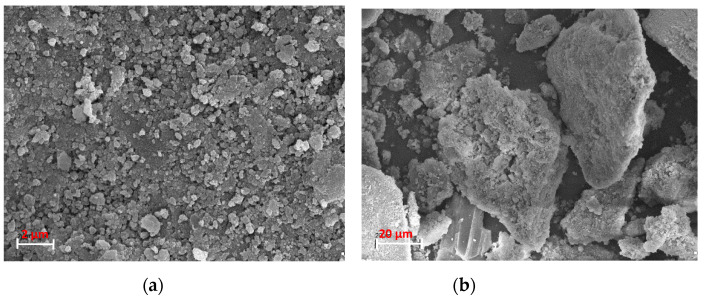
SEM images of SS-FA, scale bar: (**a**) 2 μm, (**b**) 20 μm.

The SEM analysis of SS-FA particles was performed using a scanning electron microscope, and the images are presented in Figure 5. As is seen, SS-FA is not homogeneous, and particles of different diameters and shapes are dominant. Larger particles have a more elongated, irregular shape with jagged ends. Smaller diameter particles are formed more regularly, more abundantly and have smooth tips. Spherical grains are also visible in the SS-FA. The surface of all particles is porous. Clear clusters of particles (lumps) appear in large numbers. Particle shape irregularities are the result of various parameters of the fluidizing combustion process, such as combustion duration and temperature. With the extension of the process, the obtained particles take on more spherical shapes or take a crystalline form. Similar observations could be observed in the literature [55,56,57,58]. According to the literature, the surface morphology and the specific surface area of FA particles affect the surface energy of the particles, which is responsible for the initiation of processes at the liquid–adsorbent interface. FA particles are characterized by higher surface energy and higher surface activity for a finer specific surface area. The ash porosity depends on the microporosity, the grain shape, and the extent of the grain distribution [57]. In the present study, based on the particle size distribution analysis, the SS-FA particle size was shown to be 1205 nm, the BET adsorption specific surface area was 9.105 m^2^/g, and the granulation analysis showed that the largest particle size was in the fraction with a particle size less than 0.212 μm.

### 3.2. Adsorption Analysis of Ni(II) Ions

#### 3.2.1. Analysis of pH Profile

**Figure 6:**
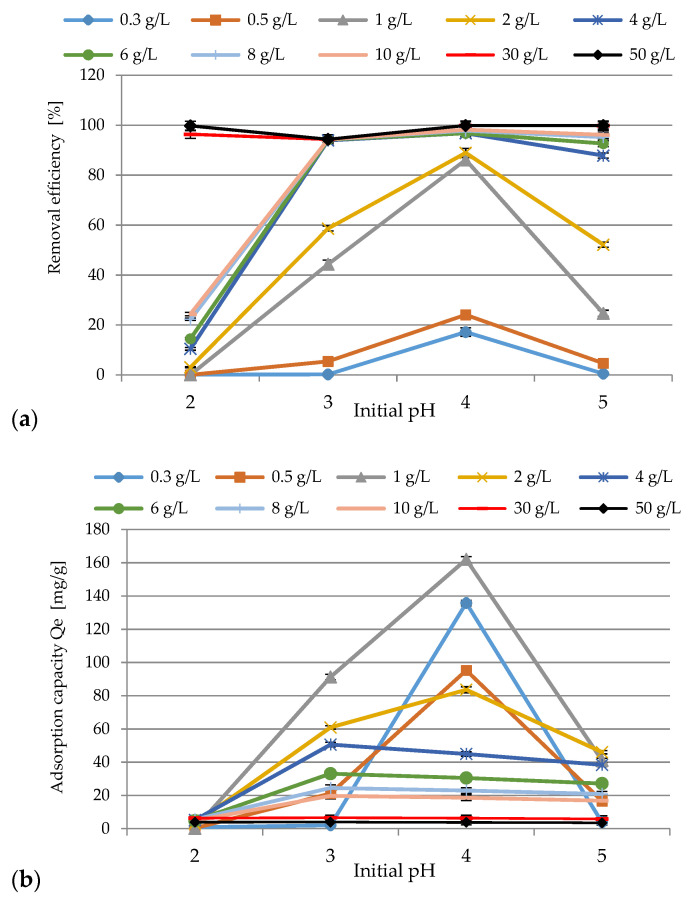
The effect of initial pH on removal efficiency (**a**) and adsorption capacity (**b**) of Ni(II) ions with SS-FA.

**Figure 7:**
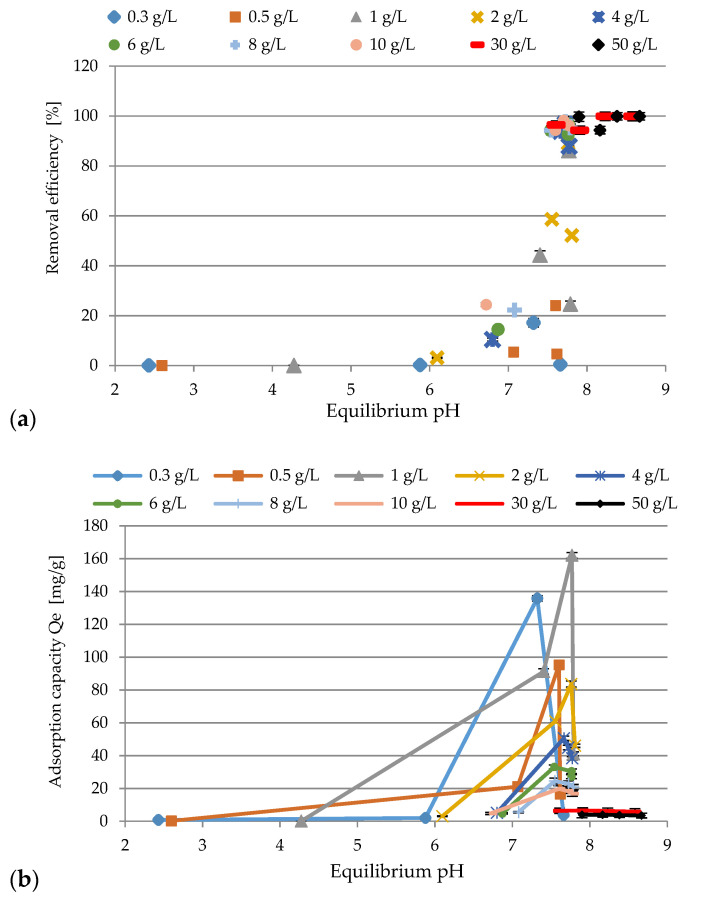
The relationship between equilibrium pH and removal efficiency (**a**) and adsorption capacity (**b**) of Ni(II) ions with SS-FA.

In the solution, pH parameter plays an important role in the adsorption process. Therefore, the influence of this factor on the efficiency of the process was investigated, and the results are presented in Figure 6a,b. The applied conditions of the experiments were as follows: the initial concentration of Ni(II) ions: 99.1 mg/L (pH 2), 105 mg/L (pH 3), 94.96 mg/L (pH 4), 88.2 mg/L (pH 5), SS-FA dosage 0.3–50 g/L, pH range of 2–5, contact time 60 min, rotation speed 200 rpm, T = 23 ± 1 °C. After analysis of the results, it was found out that the best performance was obtained at initial pH 4.0 for the adsorbent doses of 10 g/L (98.2%), 30 g/L (99.89%), 50 g/L (99.89%). In all cases an increase in adsorption was demonstrated. At initial pH 2.0, the lowest efficiency was revealed from 0.25 g/L up to 30.0 g/L, where 96.4% was achieved. To explain the effect of the initial pH of the solution on adsorption, the FA surface charge and the degree of its speciation can be taken into account. Most likely, the ion exchange mechanism was responsible for binding nickel ions. In Figure 7a,b, the relationship between equilibrium pH and removal efficiency and adsorption capacity is presented. As it is shown, any amount of SS-FA (from 0.3 to 50 g/L) increases pH up to 8.7, which is due to the alkaline nature of the adsorbent. Based on the XRD analysis, anions such as SiO_3_^2^^−^, CO_3_^2−^, SO_4_^2−^, PO_4_^3^^−^, and OH^−^ were revealed in the adsorbent mass. At higher alkaline pH, they could take part in the possible precipitation of Ni(II) ions. The SS-FA contained a lot of metal oxides, the electrostatic charge of which was affected by the pH of the solution. Hence, ion exchange and complexation processes, by binding with oxygen groups, could possibly occur. Equations (8)–(10) can represent possible general mechanism of the processes.

(8) $$-\mathrm{XOH}+{\;H}_{3}O^{+}\to {\mathrm{XOH}}_{2}^{+}+{\;H}_{2}O$$

(9) $$-\mathrm{XOH}+{\;\mathrm{OH}}^{-}\to {\mathrm{XO}}^{-}+{\;H}_{2}O$$

(10) $$2\left(-{\mathrm{XO}}^{-}\right)+{\;\mathrm{Ni}}^{2+}\to {\left(-\mathrm{XO}\right)}_{2}\mathrm{Ni}$$

where X can represent Ca, Al, K, Si, etc. In these studies, no appropriate experiments were carried out to confirm the possible mechanisms [13,59].

The surface of the SS-FA (hydrous oxide surface) was protonated by a significant amount of hydrogen ions (MOH + H^+^ → M(OH)_2_^+^), which resulted in an increase in the number of positively charged active centers and a decrease in the number of negatively charged centers. In an alkaline environment, hydrous oxides react with hydroxide ions to form deprotonated ions (MOH + OH^−^ → MO^−^ + H_2_O). In acidic solutions (initial pH < 5), nickel exists in the ionic form of Ni^2+^. Thus, it competes with H^+^ ions, which, due to electrostatic repulsion, do not favor the adsorption of positively charged nickel ions. As a result, lower adsorption was observed. When the initial pH value was adjusted to 3–4, the surface became more negatively electrostatically charged, ion exchange took place, and the appropriate groups were deprotonated, which made it possible to adsorb Ni^2+^ ions in greater amounts. The increase in adsorption with an increase in initial pH of the solution can also be explained by the presence of increased dissociation of surface hydroxyl groups from the SS-FA surface (at adsorbent dosages 30–50 g/L). At the same time, the formation of poorly soluble ions, such as NiOH^+^ and Ni(OH)_2_ may partially contribute to the maximum removal of nickel ions.

The zeta potential analysis revealed that the isoelectric point (IEP) was reached at an initial pH of 4.07. Above the value, the surface of fly ash seemed to be negatively charged. The adsorption experiments indicated that under optimal conditions at initial pH 4, the adsorption efficiency was the highest. At initial pH 5, the adsorption began to decrease. The phenomenon can be explained in this way: that repulsive electrostatic forces started to be dominant in the solution. Three different mechanisms can be proposed for the impact of the surface charge. At initial pH < 5, competitive adsorption of Ni^2+^ with H^+^ and H_3_O^+^ ions on SS-FA surface probably occurred. At initial pH range 5–6, partial adsorption and surface precipitation of NiO and Ni(OH)_2_ took place. At initial pH > 6, precipitation of nickel hydroxides predominated. The neutral NiO and Ni(OH)_2_ particles were no longer attracted by the adsorbent particles, and electrostatic repulsive forces were acting [60,61].

#### 3.2.2. Effect of Adsorbent Dosage

**Figure 8:**
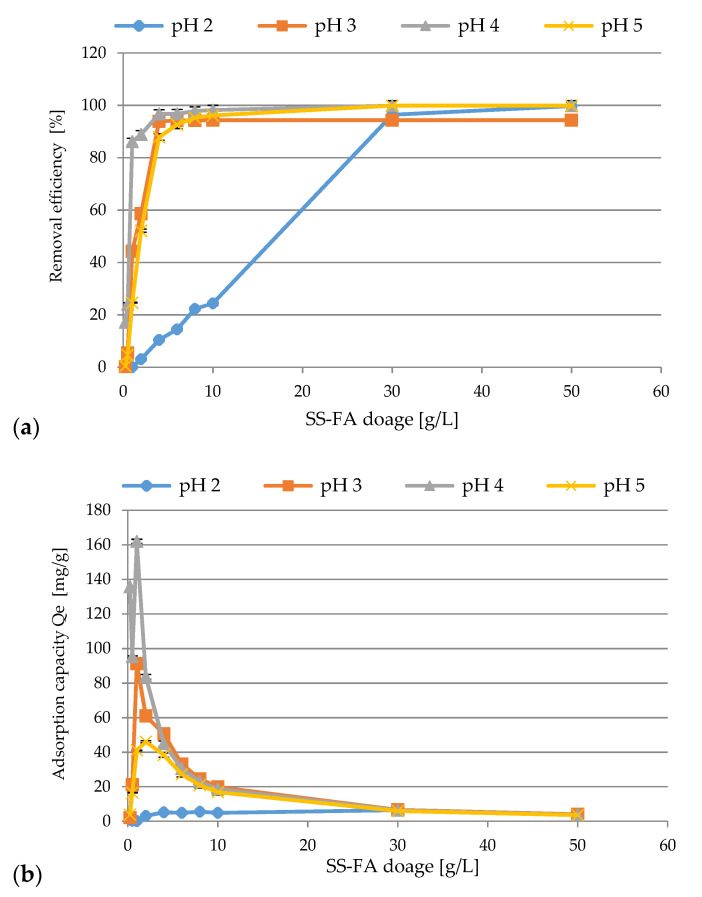
The effect of SS-FA dosage on adsorption efficiency (**a**) and adsorption capacity (**b**) of Ni(II) ions.

The effect of SS-FA dosage on the adsorption efficiency of Ni(II) ions was studied and the results are summarized in Figure 8a,b. The experiments were conducted under the following conditions: the initial concentration of Ni(II) ions: 99.1 mg/L (pH 2), 105 mg/L (pH 3), 94.96 mg/L (pH 4), 88.2 mg/L (pH 5), pH ranging from 2 to 5, rotation speed 200 rpm, T = 23 ± 1 °C, contact time 60 min, SS-FA dosage 0.25–50 g/L. Generally speaking, an increase in the removal efficiency with increasing dosage up to 50 g/L was observed. As it is seen, the dose range of 4–50 g/L could be considered optimal due to the highest efficiency in the tested pH range, and the maximum sorption results were as follows: 99.73% (pH 2, 50 g/L), 94.35% (pH 3, 50 g/L), 99.89% (pH 4, 30–50 g/L), and 99.89% (pH 5, 50 g/L). It was not necessary to increase the amount of SS-FA, as no significant changes were observed in the adsorption process under the tested conditions. Moreover, a reduction in adsorption capacity was observed starting from 161.9 mg/g (dose 1 g/L, pH 4) and ending with 3.52 mg/g (dose 50 g/L, pH 5). It was highly likely that active sites were fully occupied by Ni(II) ions when interacting with SS-FA adsorbent at their lower doses and were not fully utilized at higher doses. The decrease in sorption capacity is the result of an increase in the mass of the adsorbent, which resulted in an increase in the number of available metal ion binding sites.

#### 3.2.3. Effect of Initial Concentration of Ni(II) Ions

**Figure 9:**
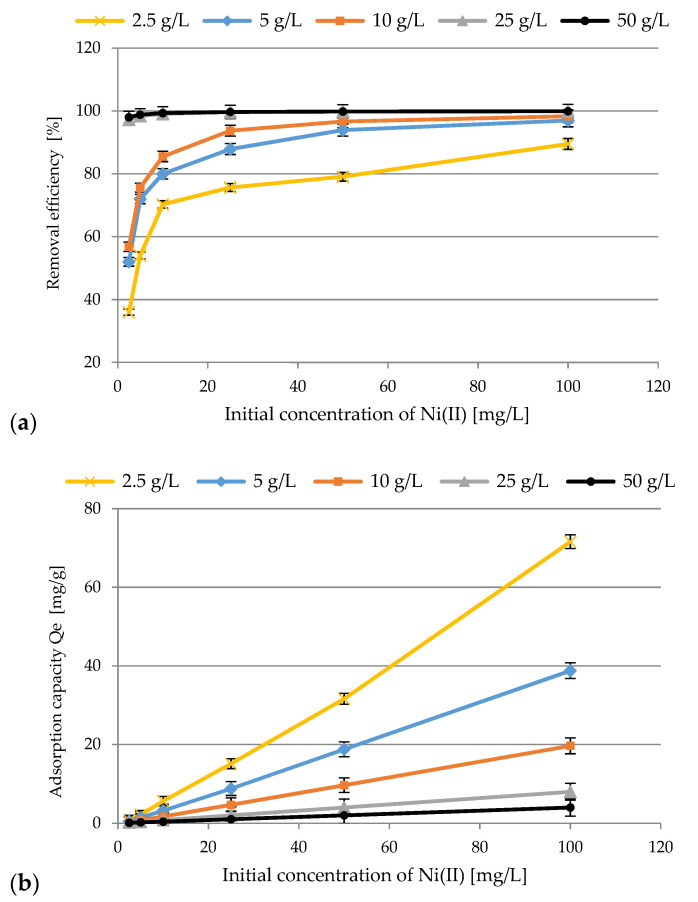
The effect of initial concentration of Ni(II) ions on SS-FA removal efficiency (**a**) and adsorption capacity (**b**).

Adsorption behavior under different initial concentration of Ni(II) ions was studied, and the results are presented in Figure 9. After analyzing the previous research results, it was decided to apply the following experimental conditions: initial concentration of Ni(II) ions (2.5–100 mg/L), adsorbent dosages 2.5–50 g/L, initial pH 4, contact time 60 min, rotation speed 200 rpm, T = 23 ± 1 °C. Generally speaking, an increase in adsorption efficiency was observed. The highest removal equal to 99.9% was achieved for the SS-FA dosage of 50 g/L and initial concentrations of 50 and 100 mg/L. Furthermore, a constant increase in the adsorption capacity ranging from the minimum values (0.09–3.99 mg/g) to the maximum value of 71.6 mg/g was observed.

Based on this analysis of the adsorption process, it was found out that the initial concentration of nickel ions influences the saturation of the SS-FA surface. As shown in Figure 9, the initial Ni(II) concentration was sufficient to initiate ion exchange at the interface between the aqueous and solid phase. Higher metal ion removal efficiency is proportional to greater driving force of mass transfer and lower resistance to metal sorption. Based on the literature, Ni(II) ions have an ionic radius equal to 70 pm. Many studies show that at the molecular level, the smaller the ionic radius of a metal, the greater its propensity to hydrolyze in aqueous solutions. Hydrolyzed molecules have a lower ability of adsorption, which translates into a decrease in the efficiency of the adsorption process [13,62,63].

#### 3.2.4. Kinetics Analysis

##### Studies of Contact Time

**Figure 10:**
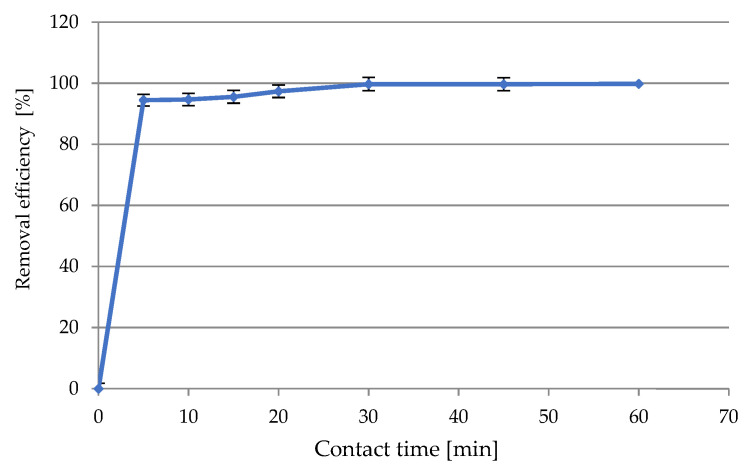
The effect of contact time on the Ni(II) ion removal efficiency.

The influence of contact time on adsorption was investigated, and the results are presented in Figure 10. This parameter is important for the effective use of adsorbents in industry. Determination of the optimal contact time in the process can be successfully used to determine the amount of solutions in adsorption processes, to design them, and also to effectively reduce the costs of the process. Our previous research has helped to establish the following optimal experimental conditions: initial concentration of Ni(II) 100 mg/L, initial pH 4.0, SS-FA dosage 30 g/L, rotation speed 200 rpm, T = 23 ± 1 °C. The maximum sorption (99.71%) was obtained within the first 30 min of the process, and there were no significant changes until 60 min. The rapid initial increase in adsorption could have been caused by the use of optimal process conditions, but also by the high concentration of Ni(II) ions at the interface and the availability of more free active sites on the surface of the material. The mechanism of the ion exchange reaction can take many forms, but the equilibrium of the process was achieved gradually by the occupation of active centers by cations.

##### Pseudo-First-Order and Pseudo-Second-Order Kinetic Models

**Figure 11:**
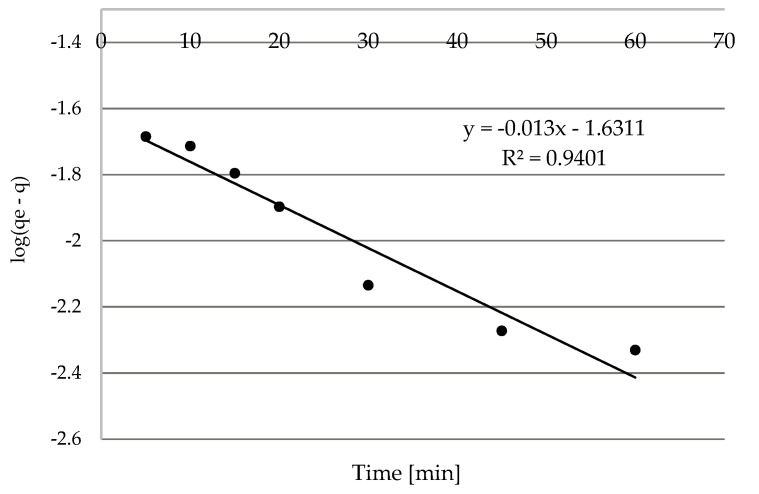
The pseudo first-order kinetic plot for the adsorption of Ni(II).

**Figure 12:**
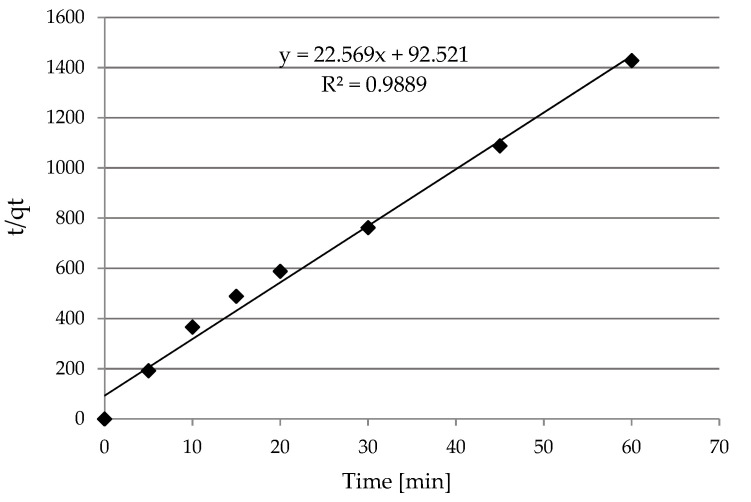
The pseudo second-order kinetic plot for the adsorption of Ni(II).

Kinetics of the sorption of Ni(II) ions on the SS-FA was analyzed. Hence, pseudo-first order and pseudo-second order models were used (Figure 11 and Figure 12), and the determined kinetic parameters are shown in Table 3. On the basis of the calculated values, it was found that a higher correlation coefficient *R^2^* was obtained for the pseudo-second-order model. Hence, the result suggests that this model is a better fit for the description of the kinetics of the adsorption process. This means a greater degree of correlation between the experimental *q_e_* and calculated *q_t_* values. Therefore, the sorption process took place by diffusion and could probably take the form of chemisorption. Nickel ions could form chemical bonds and have an affinity for active sites that increased their coordination number with the surface, as a result of which adhesion to the sorbent surface occurred. The research showed that the particles were less likely to collide with each other under the conditions of a lower concentration of nickel ions in the aqueous solution. An additional effect was faster binding of Ni(II) ions with active centers on the surface of the material.

#### 3.2.5. Isothermal Analysis

In these studies, the adsorption process was analyzed using Langmuir and Freundlich isotherms models, and the figures are presented in the Supplementary Material (Figures S5 and S6). Based on the calculated parameters, it was found that the adsorption data was better suited to the Freundlich model (Table 4). According to the equation, *K_L_* constant describes the spontaneity of adsorption and relates to the sorbent and binding energy of the solute. The spontaneity of the adsorption reaction increases proportionally with the increase in the *K_L_* value. This dependence translates into the presence of a more stable adsorbent and greater efficiency of the process. Nevertheless, the Freundlich isotherm describes the relationship between the concentration of metal ions dissolved in a liquid at equilibrium (*Ce*) and the concentration of dissolved ions on the surface of an adsorbent (*qe*). A comparison of maximum adsorption capacity with other selected adsorbents is shown in Table 5.

### 3.3. FT-IR Studies

**Figure 13:**
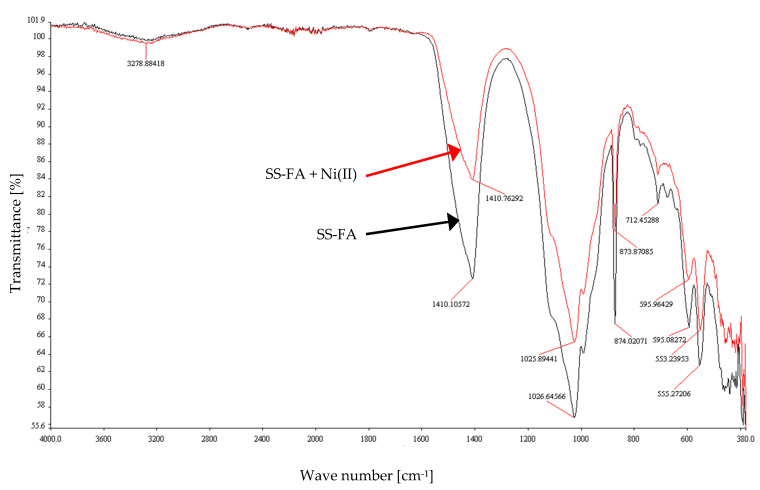
FT-IR spectrum of SS-FA before and after Ni(II) adsorption.

The FT-IR analysis of SS-FA before and after the removal process was conducted, and the spectra are presented in Figure 13. To this purpose, the samples were analyzed under the following experimental conditions: SS-FA dose 30 g/L, initial concentration of Ni(II) ions 10 mg/L, initial pH 4, T = 23 ± 1 °C, contact time 60 min. The explanation of the most essential FT-IR peaks is presented in Table 6. The spectra were analyzed before and after the adsorption process in terms of differences in shape, intensity of bands, frequency, and possible interaction of functional groups with Ni(II) ions. Figure 13 shows that after the sorption process, the intensity of the bands shifted towards higher transmittance values, and their location remained at the same wavelengths or was slightly shifted. These changes can be identified as follows: 3278.88 (shift to 3279.15 cm^−1^), 1410.18 (shift to 1410.76 cm^−1^), 1026.64 (shift to 1025.89 cm^−1^), 873.87 (shift to 874.02 cm^−1^), 712.45 (shift to 712.8 cm^−1^), 595.08 (shift to 595.96 cm^−1^), 553.24 (shift to 555.27 cm^−1^). Bands at similar wavelengths are reported in the literature: bending O–Si–O (467 cm^−1^, silica glass and quartz), symmetric stretching Al–Si–O (571 cm^−1^), symmetric stretching Si–O–Si (796 cm^−1^), asymmetric stretching Si–O (1110 cm^−1^, 1044 cm^−1^) [84].

## 4. Conclusions

Fly ash generated from the combustion of municipal sewage sludge in the circulating fluidized bed combustion (CFBC) technology was examined for the possibility of removing Ni(II) ions from aqueous solutions. The selected analytical methods were used to evaluate the physicochemical properties of the material. The efficiency of the adsorption process depends on such parameters as the dose of the sorbent, initial pH, initial concentration, and contact time; therefore, the influence of these parameters on the process was analyzed in subsequent experiments. The conducted research showed that the maximum adsorption efficiency was 99.9% under the following conditions: initial pH 4.0, SS-FA dosage 50 g/L, initial concentration range 94.9 g/L, contact time 60 min, rotation speed 200 rpm, T = 23 ± 1 °C. Overall, however, it can be seen that the process yield was above 90% under many experimental conditions. The SS-FA material was characterized before and after adsorption by FT-IR analysis. Changes in intensity of bands and slight shifts of the peaks were observed. Additionally, the kinetics of the process was analyzed, and isotherms were determined. The analysis showed that the performed adsorption process is best described by the pseudo-second order kinetic model and the Freundlich model. The maximum adsorption capacity *q_max_* calculated on the basis of the Langmuir equation was 130.03 mg/g for an SS-FA dosage of 50 g/L.

In conclusion, as a result of the research, it was unequivocally found that post-production SS-FA waste is capable of removing Ni(II) ions with a very high process efficiency due to the content of appropriate functional groups in the material composition and favorable physicochemical properties. Promising research results are worth continuing experiments on the adsorption of other heavy metal ions. Most importantly, an essential conclusion is the real possibility of industrial use of SS-FA in order to improve water quality by removing heavy metal ions, which is in line with current global trends in sustainable development, circular economy, and climate neutrality.

## Figures and Tables

**Table 1:** Elemental composition of SS-FA (EDS microanalyzer) and comparison with the literature.

Elements	C	O	Na	Mg	Al	Si	P	S	K	Ca	Ti	Mn	Fe	Zn
Weight (%)	1.85 ± 0.06	44.79 ± 0.3	0.54 ± 0.03	3.28 ± 0.1	6.41 ± 0.3	5.51 ± 0.2	11.35 ± 0.4	0.41 ± 0.09	1.11 ± 0.1	17.68 ± 0.2	0.54 ± 0.03	0.23 ± 0.02	5.43 ± 0.3	0.87 ± 0.09
Atomic (%)	3.41 ± 0.2	61.93 ± 0.6	0.52 ± 0.03	2.99 ± 0.09	5.25 ± 0.2	4.34 ± 0.3	8.11 ± 0.3	0.28 ± 0.03	0.63 ± 0.05	9.76 ± 0.5	0.25 ± 0.02	0.09 ± 0.01	2.15 ± 0.2	0.29 ± 0.02
	Content of oxides (wt. %)													
References	CO_2_		Na_2_O	MgO	Al_2_O_3_	SiO_2_	P_2_O_5_	SO_3_	K_2_O	CaO	TiO_2_	MnO	Fe_2_O_3_	ZnO
This research	6.97 ± 0.3		0.73 ± 0.04	5.44 ± 0.2	12.11 ± 0.6	11.78 ± 0.6	26.02 ± 0.8	1.02 ± 0.1	1.33 ± 0.1	24.74 ± 0.8	0.89 ± 0.03	0.29 ± 0.01	7.77 ± 0.3	1.08 ± 0.08
[48]	―		0.37	1.4	27.0	48.8	1.2	0.22	0.85	6.2	1.3	0.15	10.2	―
[49]	―		0.37	0.77	26.49	53.36	1.43	0.20	0.80	1.34	1.47	―	10.86	―
[50]	―		―	0.97	22.03	57.25	―	0.76	0.52	2.97	0.68	―	8.36	―
[51]	―		0.38	1.2	23.63	―	1.31	0.28	0.84	1.74	1.32	0.13	15.3	―
[52]	―		0.42	0.78	23.59	52.11	1.31	0.49	0.80	2.61	0.88	0.03	7.39	―
[53]	―		0.61	0.3	29.8	56.01	0.44	―	0.73	2.36	1.75	―	3.58	―

**Table 2:** Adsorption and desorption surface parameters of SS-FA.

Parameters	Values
BET adsorption cumulative surface area (S_BET_) (m^2^/g)	9.105
BET desorption cumulative surface area (S_BET_) (m^2^/g)	12.368
BJH adsorption cumulative volume of pores (Vpa) (cm^3^/g)	0.032417
BJH desorption cumulative volume of pores (Vpd) (cm^3^/g)	0.03302
BJH adsorption average pore diameter (Apda) (nm)	14.2408
BJH desorption average pore diameter (Apdd) (nm)	10.6791

**Table 3:** Adsorption parameters of pseudo-first-order and the pseudo-second-order rate equations.

Metal Ion	Adsorbent Dosage [g/L]	Pseudo-First-Order Kinetic Model			Pseudo-Second-Order Kinetic Model		
		*k_ad_* [min^−1^]	*q_e_* [mg/g]	*R^2^*	*k* [g/mg min]	*q_e_* [mg/g]	*R* ^2^
Ni(II)	30	0.030	0.023	0.940	2441.91	0.044	0.989

**Table 4:** Isotherm model parameters for adsorption of Ni(II) using SS-FA.

Metal Ion	Adsorbent Dosage (g/L)	Langmuir Isotherm			Freundlich Isotherm		
		Calculated *q_m_* (mg/g)	*K_L_* (L/mg)	*R* ^2^	*K_f_* (mg/g) (L/mg)^(1/n)^	*n*	*R* ^2^
Ni(II)	2.5	94.72	0.0297	0.951	2.489	0.827	0.974
	5	96.8	0.0208	0.946	1.954	0.745	0.958
	10	81.81	0.034	0.977	3.152	0.869	0.980
	25	126.32	0.1166	0.992	21.169	0.902	0.995
	50	130.03	0.0435	0.983	11.242	0.820	0.984

**Table 5:** Comparison of maximum adsorption capacity of SS-FA with different adsorbents.

Adsorbents	Adsorption Capacity (mg/g)	Ref.
Carbon nanotubes–granular activated carbon (CNT–GAC)	0.07	[64]
Nano crystalline hydroxyapatite (nano HAp)	2.28	[65]
Graphene	10.8	[66]
LD slag	15	[67]
Multi-walled carbon nano tubes (MWCNT)	17.86	[68]
Graphene/MnO_2_	46.55	[66]
Dolochar ash geopolymer	48	[69]
Pyrophyllite-based geopolymer	49	[70]
Hollow fibers	62.51	[71]
Graphene oxide/carboxy methyl cellulose	72.04	[72]
Nanostructured Al_2_O_3_	83.33	[73]
Ion-imprinted polymer	86.3	[74]
MgO nanosheets (ultrasonic method)	87	[75]
Polyamidoxime chelating resin (PAO-AN) three dimensional	130	[76]
Sewage sludge fly ash (SS-FA)	130.03	This study
Graphene oxide (GO)	178	[77]
Graphene oxide modified with 2,20-dipyridylamine (GO-DPA)	180.89	[78]
MgO nanosheets (precursor calcination)	185.5	[79]
Activated carbon derived from *Xanthoceras*	188	[80]
Polyvinyl alcohol/CNTs nanoporous architectures (3DPCA)	225.6	[81]
Slag based geopolymer	414	[82]
Sieved geopolymer sample (SGS)	520	[83]

**Table 6:** FT-IR peaks of SS-FA.

FT-IR Band (cm^−1^)	Assignment (Vibrations, Species)
3278.88	stretching vibrations O–H
1410.76, 1410.1	valence vibration of carbonate ions
1025.89, 1026.64	asymmetric stretching vibrations of silica Si–O–Si
873.87, 874.02	symmetric stretching of Al–O–M, vibration of carbonates (calcite)
712.45	symmetric stretching of Si–O–Si and Al–O–Si
595.96, 595.08	stretching vibrations Al–O, Si–O–M
553.24, 555.27	O–P–O, O=P–O bending vibration (probably P_2_O_5_)
380–440	bond bending vibrations Si–O–Si

## Data Availability

Data is contained within the article.

## Supplementary Materials

The following are available online at https://www.mdpi.com/article/10.3390/ma14113106/s1, Figure S1. Particle size distribution of fly ash (SS-FA) determined by laser diffraction; Figure S2. The low temperature BET adsorption and desorption isotherm; Figure S3. Linear form of BET adsorption isotherm; Figure S4. Pore volume distribution (the BJH method); Figure S5. Langmuir isotherms for adsorption of Ni(II) ions with SS-FA; Figure S6. Freundlich isotherms for adsorption of Ni(II) ions with SS-FA.
