# Supplementary material for: Highly Effective Adsorption Process of Ni(II) Ions with the Use of Sewage Sludge Fly Ash Generated by Circulating Fluidized Bed Combustion (CFBC) Technology
Source: Materials (Basel). 2021 Jun 5;14(11):3106. doi: 10.3390/ma14113106 (PMC8201213; doi:10.3390/ma14113106)
Supplement: Supplementary file 1 [file materials-14-03106-s001.zip › materials-1210900 - SM-for pub.pdf]

materials

MDPI

Supplementary Material

Highly Effective Adsorption Process of Ni(II) Ions with the Use of Sewage Sludge Fly Ash Generated by Circulating Fluidized Bed Combustion (CFBC) Technology

Tomasz Kalak *, Kinga Marciszewicz and Joanna Piepiórka-Stepuk

Methods of sunflower wood fly ash characterization

In the research, sunflower wood fly ash (SW-FA) particles with a diameter in the range of 0 - 0.212 mm were used. Firstly, physical and chemical properties of the material were analyzed using several methods, including:

1) Determination of granulation was performed by a sieve method in accordance with the standard PN-C-97555-01:1988P. In order to separate individual ash fractions, they were screened through four sieves with mesh diameters of 0.212 to 1.0 mm over 1 hour. Fractions retained on individual sieves were weighed and the procedure was performed in triplicate. The main composition was percentage content of particles of a certain diameter (grain faction, X [%]) was calculated according to the Equation (1).

$$X = \frac{m_1 \times 100\%}{m_2} \tag{1}$$

where: m_1 [g] is mass of sifted material and m_2 [g] - initial mass of a sample.

2). Determination of bulk density was performed according to the standard PN-S-96035:1997. The mass and volume of fly ash was determined using a measuring cylinder. Measurements were repeated six times. Bulk density X [g/cm^3] was calculated according to the Equation (2).

$$X = \frac{m_1 - m_0}{V} \tag{2}$$

where: m_1 [g] is mass of a cylinder with fly ash; m_0 [g] - mass of an empty cylinder and V [cm^3] - volume of fly ash in a cylinder.

3) Determination of particle size distribution was performed by the laser diffraction method using a Zetasizer Nano ZS (Malvern Instruments Ltd., United Kingdom), which is capable of measuring powders with a size distribution ranging from 0.2 to 2000 μm.

4) The elemental composition and mapping of fly ash samples was examined with a scanning electron microscope (SEM) Hitachi S-3700N with an attached a Noran SIX energy dispersive X-ray spectrometer (EDS) microanalyser (ultra-dry silicon drift type with resolution (FWHM) 129 eV, accelerating voltage: 20.0 kV).

5) X-ray diffraction measurements were made using Bruker AXS D8 Advance (Germany). In configuration the diffractometer is equipped with Johansson monochromator (λCu K$_{\alpha 1}$ = 1,5406 Å) and silicon strip detector LynxEye. The minimum measurement angle is 0,6° 2Θ deg. The XRD powder diffraction method needs delivered sample to be carefully powderized. A standard measuring dish has container for powder with diameter ca. 25 mm and ca. 1.5 mm deepth. Before measurement sample powder needs to be mildly pressed.

6) Thermal stability was determined by thermogravimetric analysis using the apparatus Setsup DTG, DTA 1200 (Setram). Fly ash samples were heated at the speed

10°C/min. in the temperature range 30 - 1000°C under nitrogen atmosphere at a flow rate of 20 mL/min.

7) The specific surface area and the average pore diameter were determined with the Brunauer, Emmett and Teller (BET) method using Autosorb iQ Station 2 (Quantachrome Instruments, USA).

8) The pore volume was determined by Barret, Joyner and Halenda (BJH) method using Autosorb iQ Station 2 (Quantachrome Instruments, USA).

9) Investigation of electrokinetic zeta potential was carried out using Zetasizer Nano ZS (Malvern Instruments Ltd., United Kingdom) equipped with autotitrator (MPT-2 Autotitrator). The apparatus uses a combination of electrophoresis and laser particle movement measurement based on the Doppler effect. The instrument measures the rate of particle movement in the liquid after switching on the electric field. The speed of motion of the particle is defined as its electrophoretic mobility, which is automatically calculated and converted to the zeta potential using the Smoluchowski's Equation (3).

$$\zeta = \frac{4\pi\eta}{\varepsilon} U \quad (3)$$

where: ζ is zeta potential; π - the constant; η - the viscosity of the suspending liquid; ε - the dielectric constant and U - electrophoretic mobility. Ash samples were dispersed in distilled water and pH of the slurry was adjusted by addition 0.2 M HCl and 0.2 M KOH before measurements at room temperature (23±1°C) of electrophoretic mobility of particles.

10) The morphology of the fly ash samples was examined with a scanning electron microscope (SEM) EVO-40 (Carl Zeiss, Germany).

11) The surface structure of fly ash was examined in infrared spectroscopy using a Fourier transform attenuated total reflection (FT-IR ATR) Spectrum 100 (Perkin-Elmer, Waltham, USA).

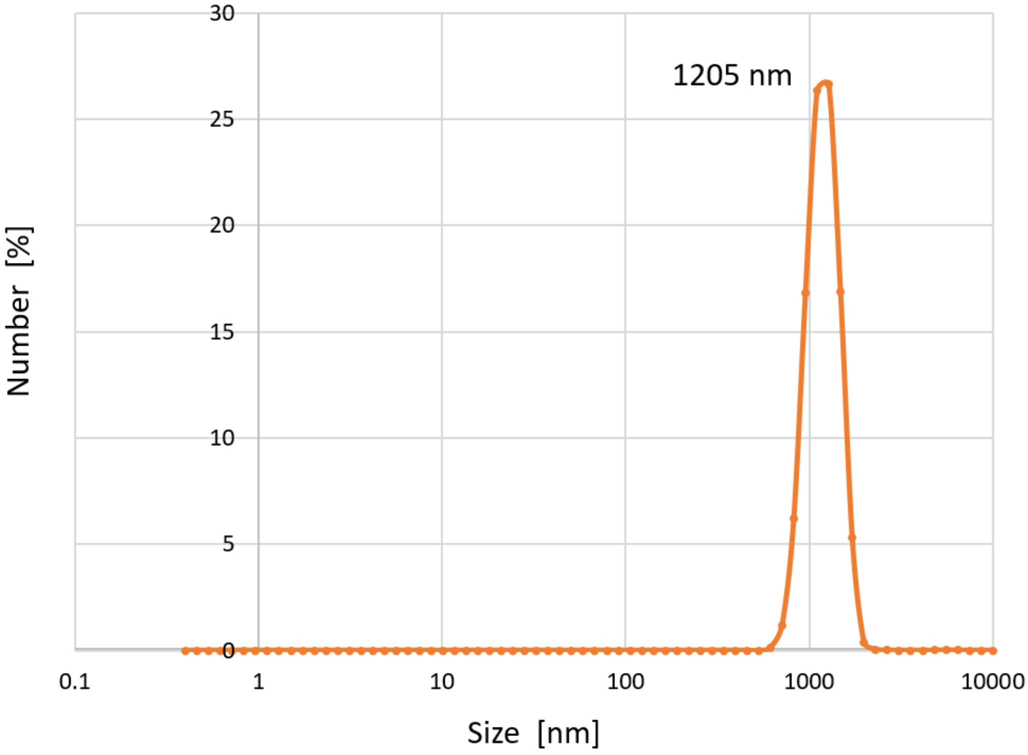

Figure S1. Particle size distribution of fly ash (SS-FA) determined by laser diffraction.

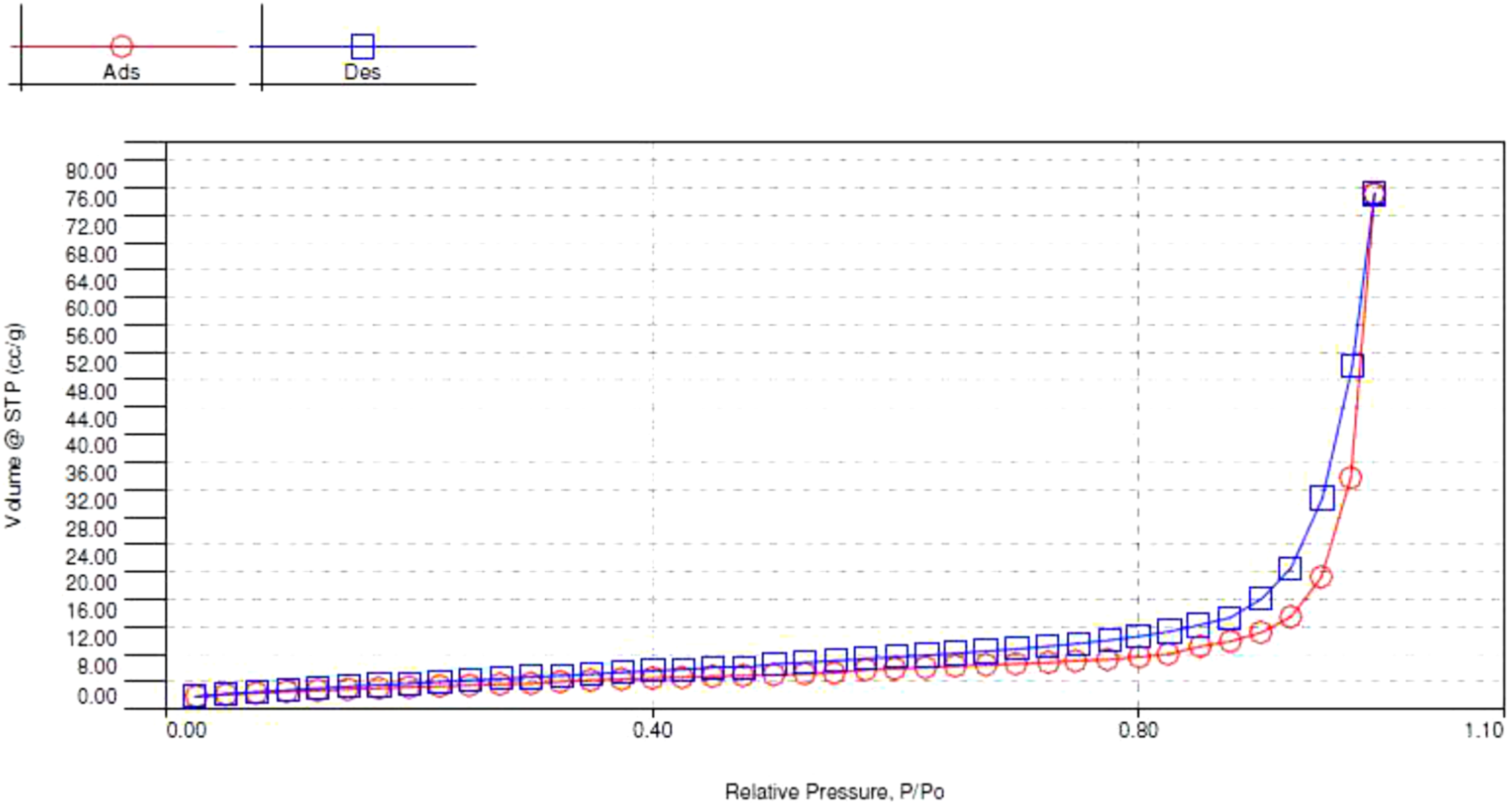

Figure S2. The low temperature BET adsorption and desorption isotherm.

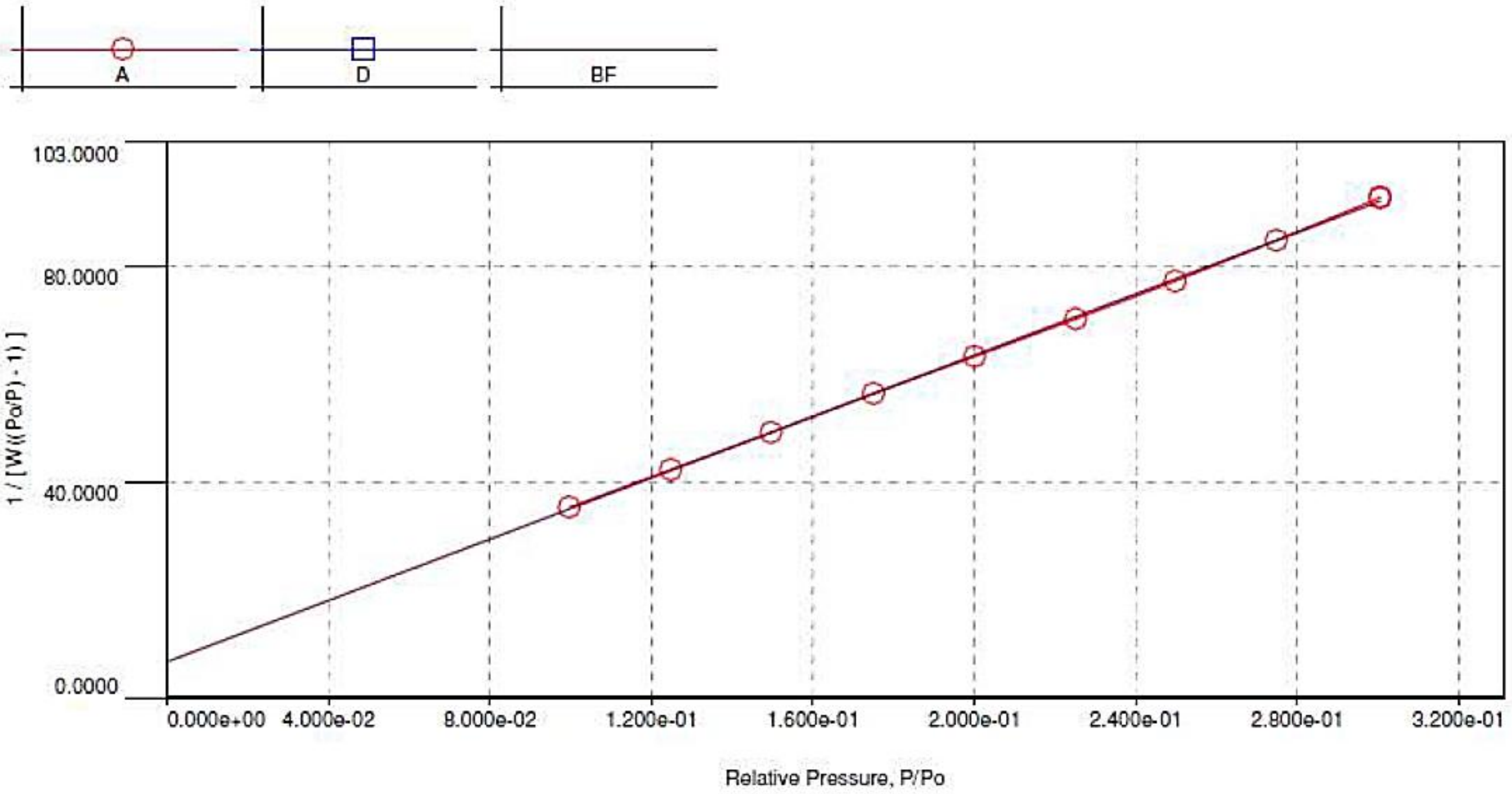

Figure S3. Linear form of BET adsorption isotherm.

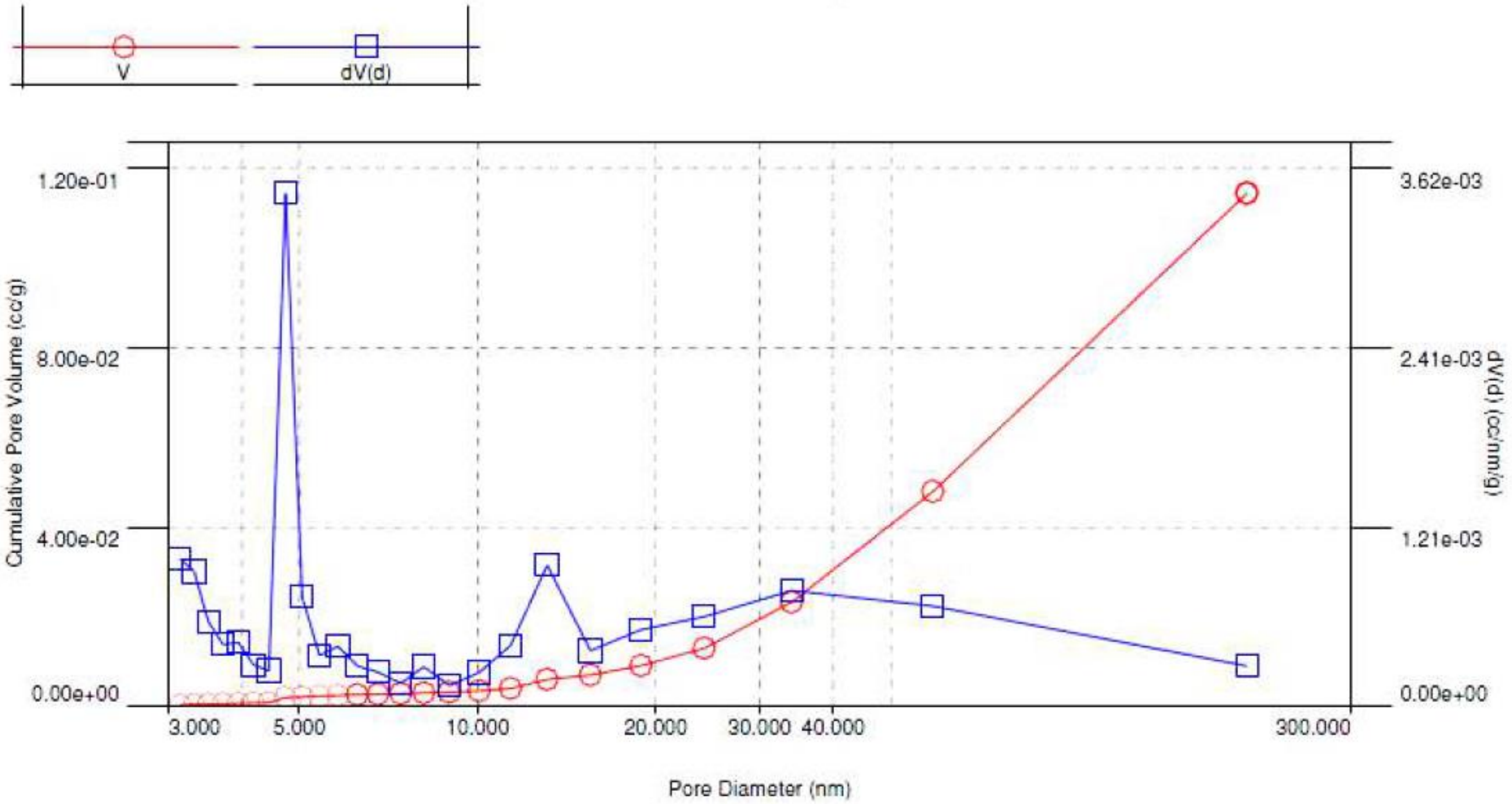

Figure S4. Pore volume distribution (the BJH method).

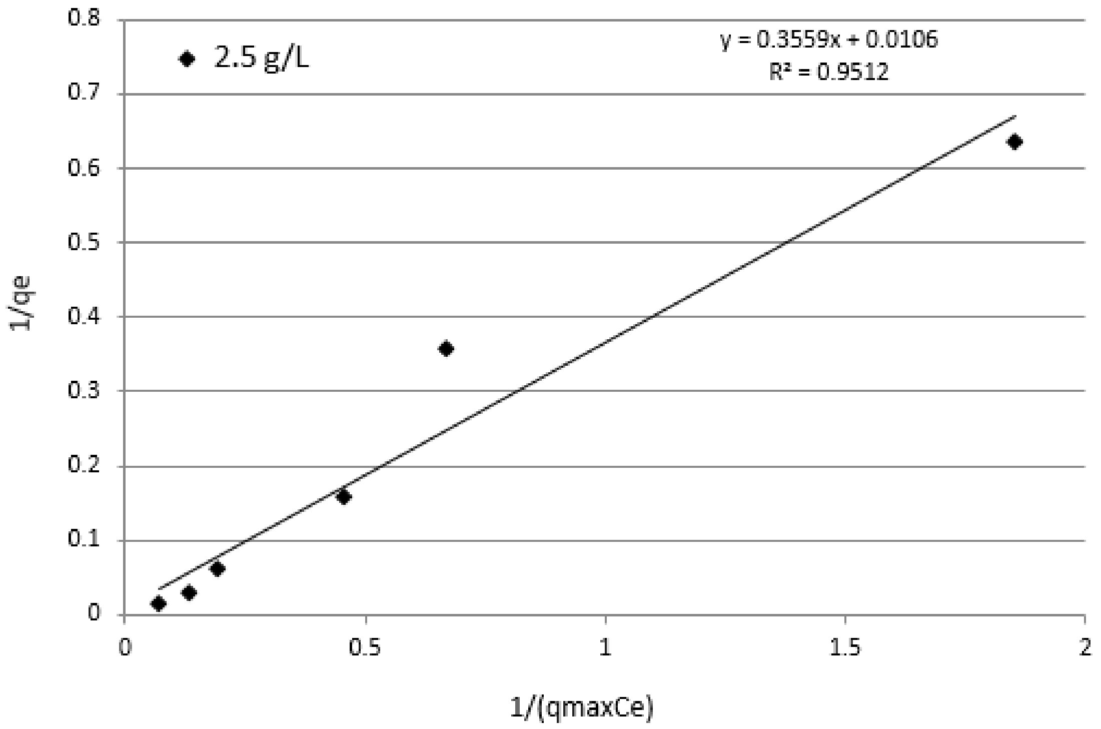

2.5 g/L
y = 0.3559x + 0.0106
R² = 0.9512
1/qe
1/(qmaxCe)
0.8
0.7
0.6
0.5
0.4
0.3
0.2
0.1
0
0
0.5
1
1.5
2

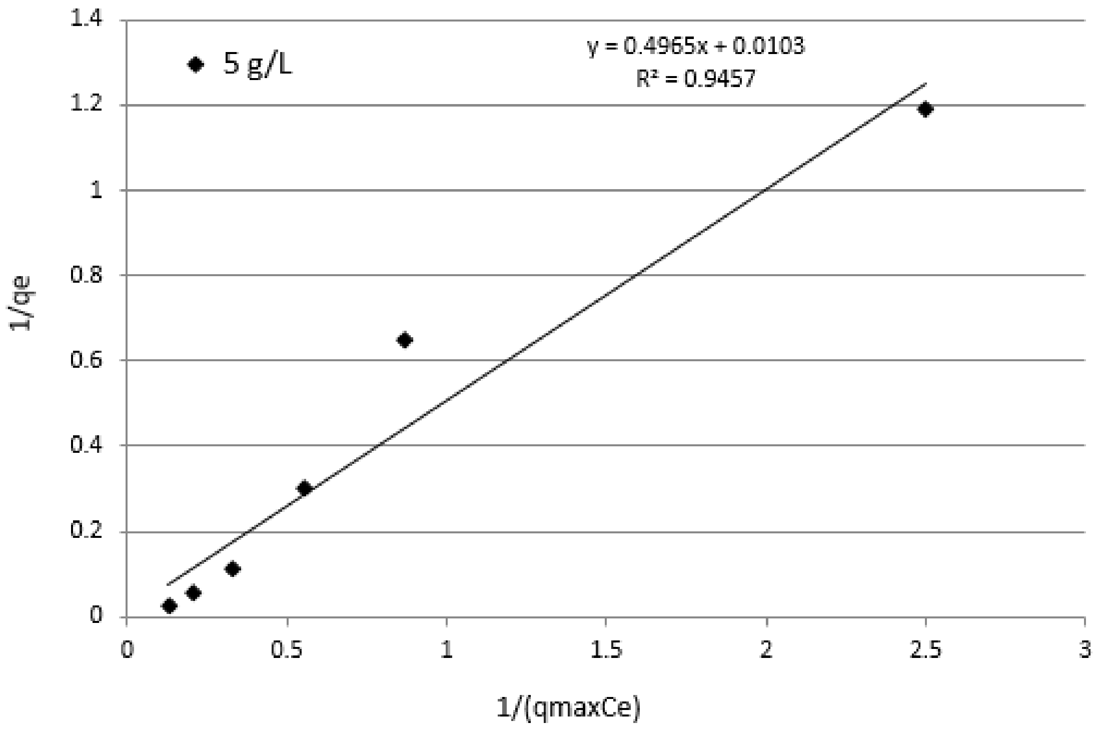

5 g/L
y = 0.4965x + 0.0103
R² = 0.9457
1/qe
1/(qmaxCe)
1.4
1.2
1
0.8
0.6
0.4
0.2
0
0
0.5
1
1.5
2
2.5
3

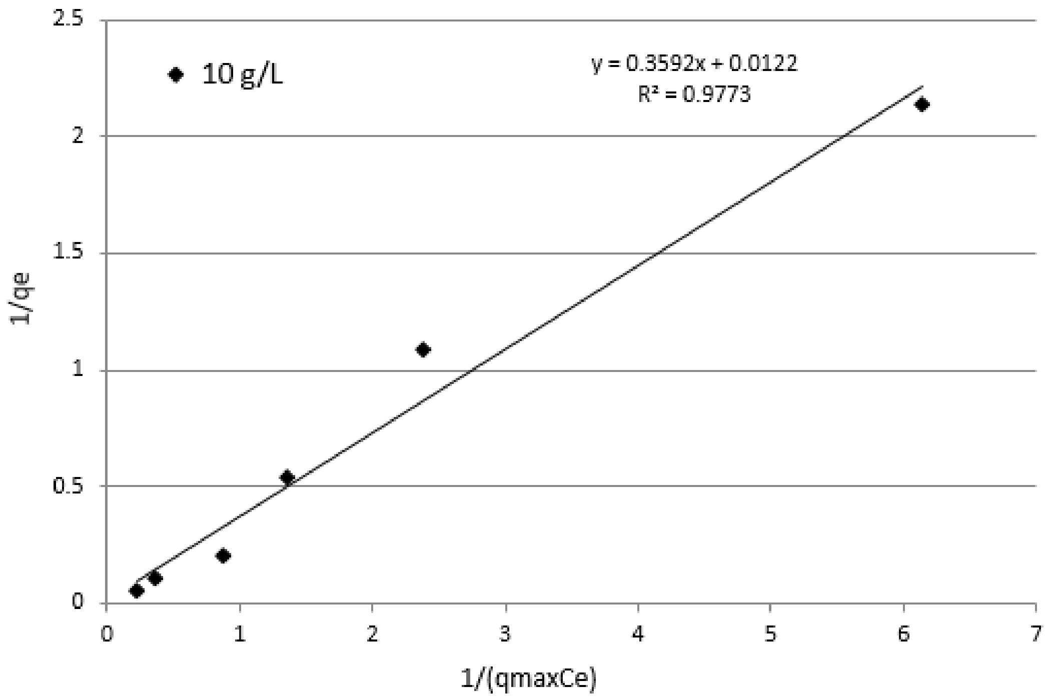

10 g/L
y = 0.3592x + 0.0122
R² = 0.9773
1/qe
1/(qmaxCe)

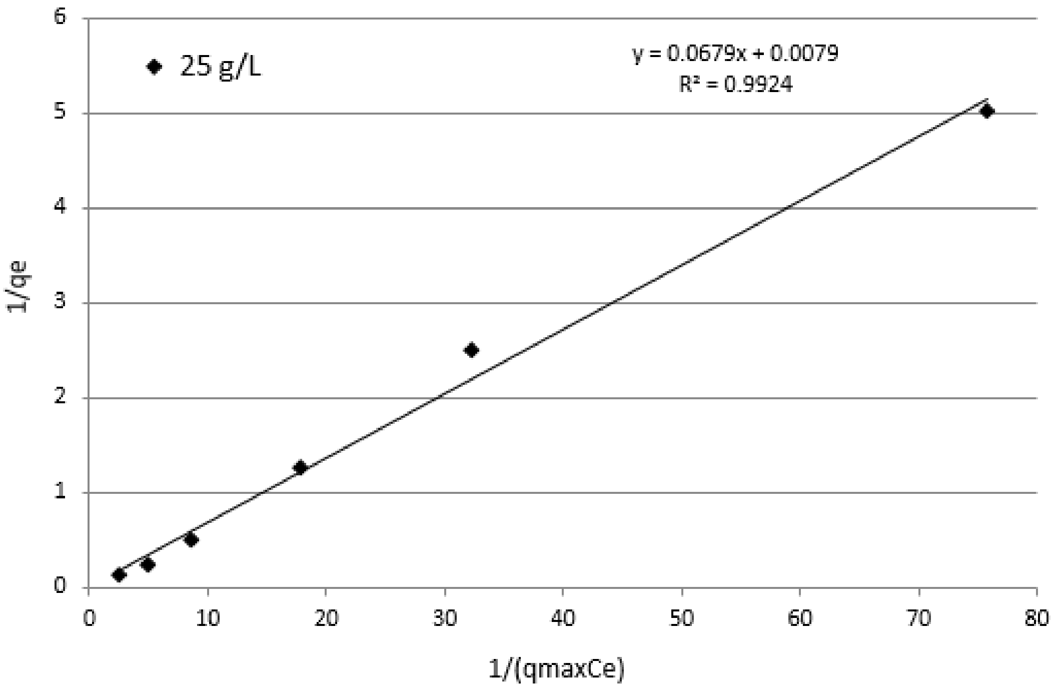

25 g/L
y = 0.0679x + 0.0079
R² = 0.9924
1/qe
1/(qmaxCe)

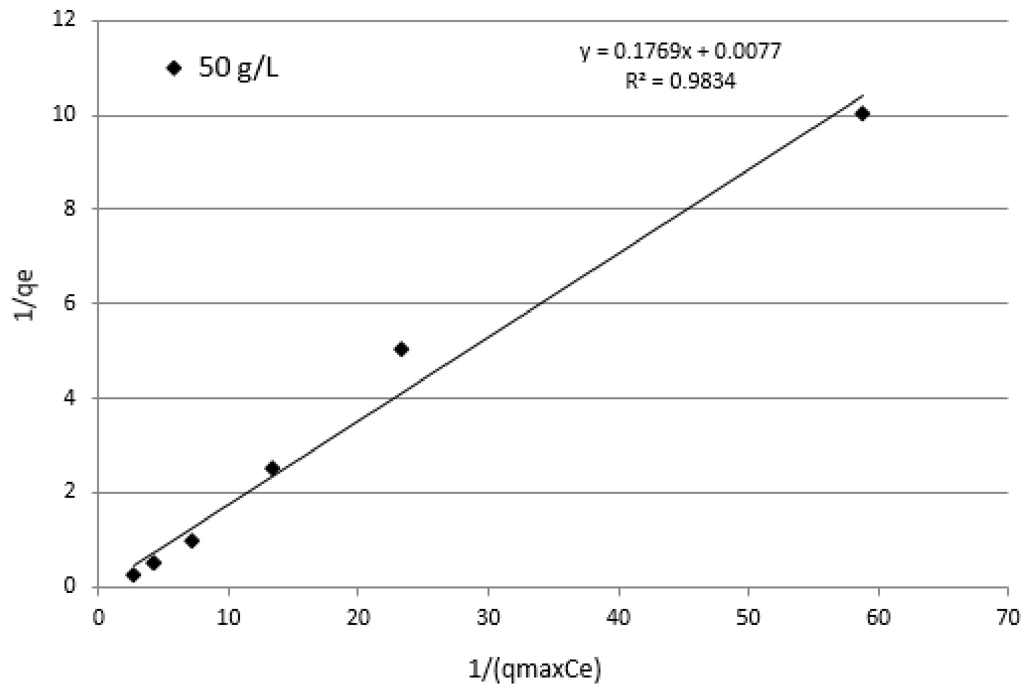

Figure S5. Langmuir isotherms for adsorption of Ni(II) ions with SS-FA (adsorbent dosage 2.5 - 50 g/L, initial pH 3.97, initial concentration of Ni(II) 2.5 – 100 mg/L, agitation speed 200 rpm, T = 23±1 °C).

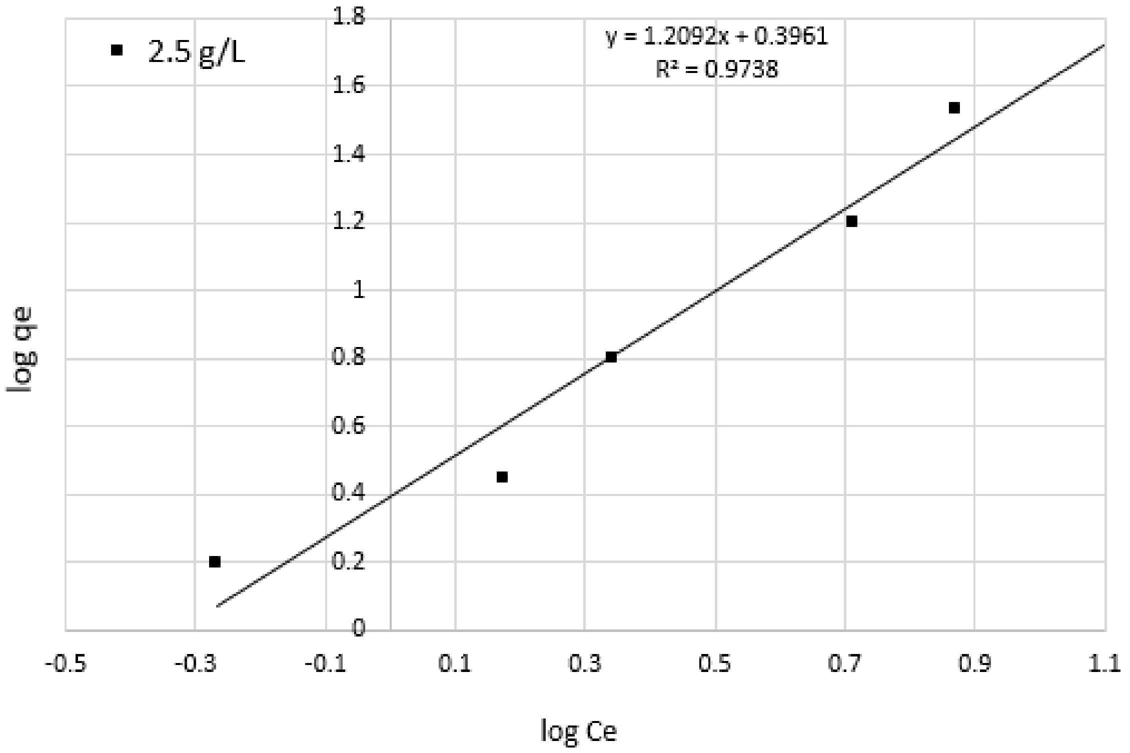

2.5 g/L
y = 1.2092x + 0.3961
R² = 0.9738
log qe
log Ce
1.8
1.6
1.4
1.2
1
0.8
0.6
0.4
0.2
0
-0.5
-0.3
-0.1
0.1
0.3
0.5
0.7
0.9
1.1

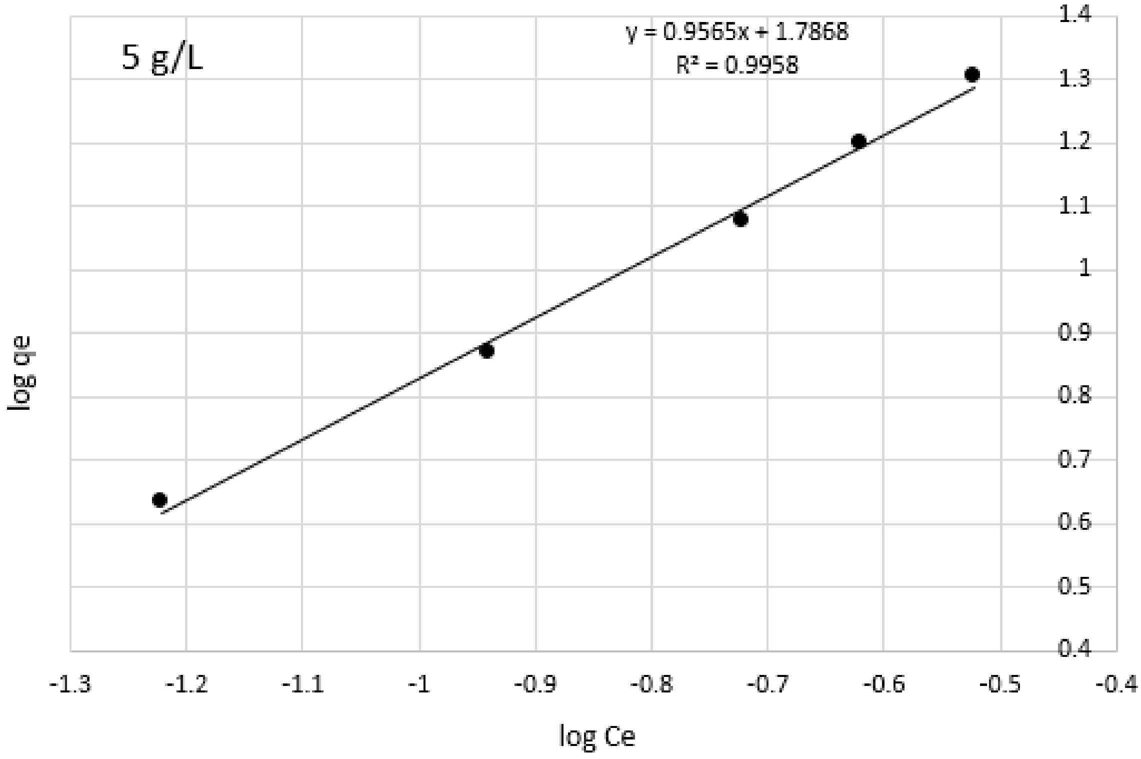

5 g/L
y = 0.9565x + 1.7868
R² = 0.9958
log qe
log Ce
1.4
1.3
1.2
1.1
1
0.9
0.8
0.7
0.6
0.5
0.4
-1.3
-1.2
-1.1
-1
-0.9
-0.8
-0.7
-0.6
-0.5
-0.4

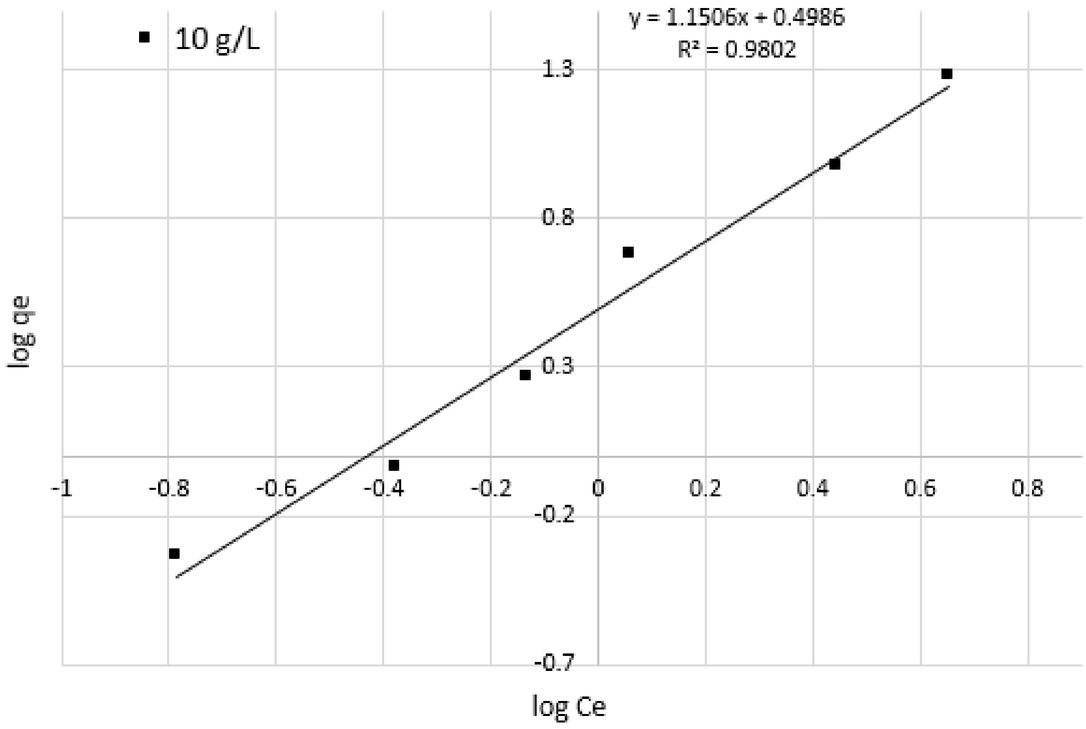
10 g/L
y = 1.1506x + 0.4986
R² = 0.9802
1.3
0.8
0.3
-0.2
-0.7
-1
-0.8
-0.6
-0.4
-0.2
0
0.2
0.4
0.6
0.8
log qe
log Ce

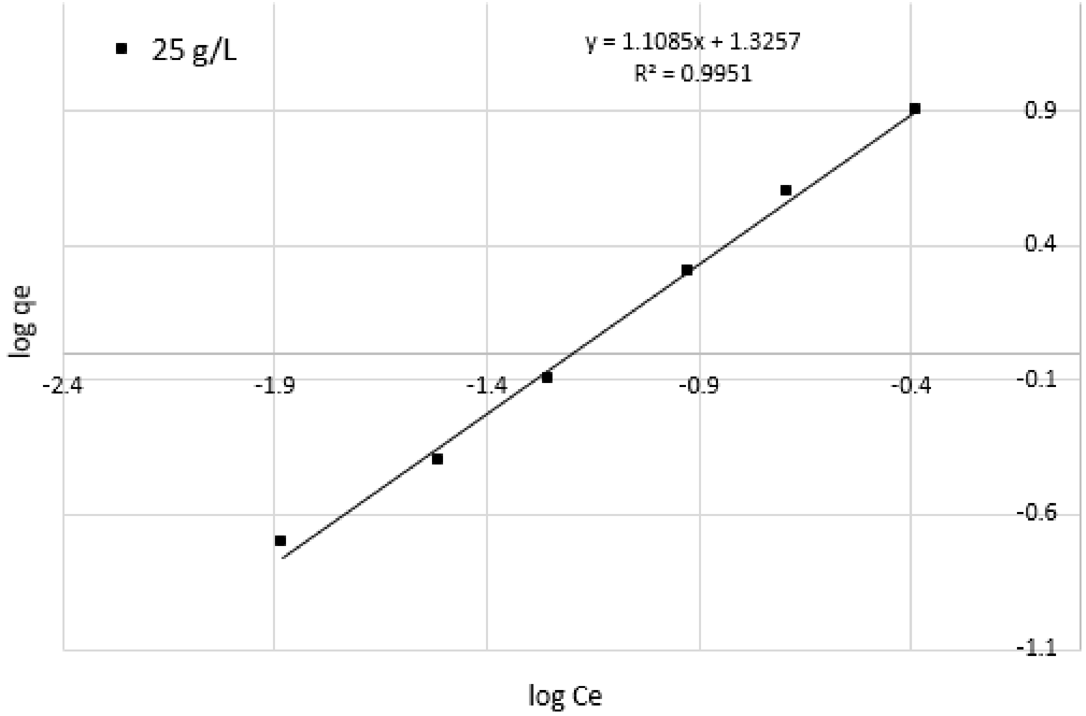
25 g/L
y = 1.1085x + 1.3257
R² = 0.9951
0.9
0.4
-0.1
-0.6
-1.1
-2.4
-1.9
-1.4
-0.9
-0.4
log qe
log Ce

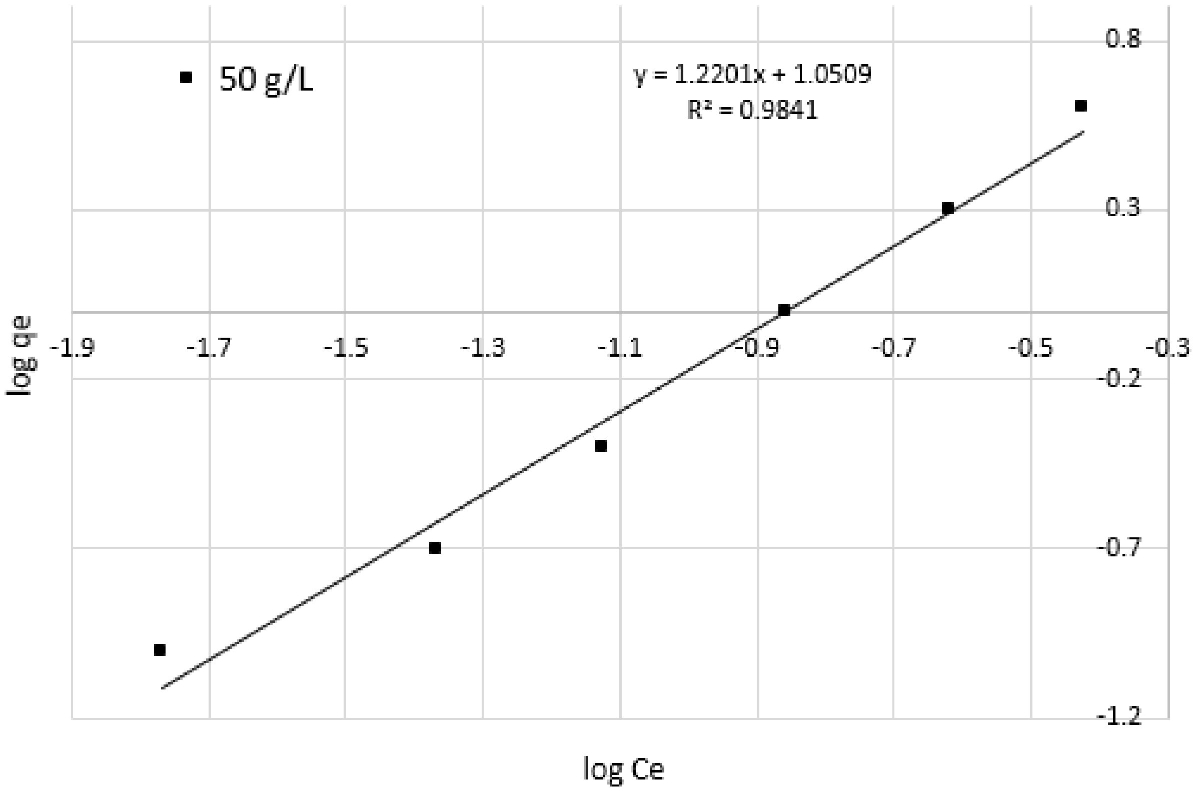

Figure S6. Freundlich isotherms for adsorption of Ni(II) ions with SS-FA (adsorbent dosage 2.5 - 5 g/L, initial pH 3.97, initial concentration of Ni(II) 2.5 – 100 mg/L, agitation speed 200 rpm, T = 23±1 °C).
